# The evolutionarily conserved C-terminal tail of the Dib1 protein is required for spliceosome efficacy and Dib1 protein stability

**DOI:** 10.1016/j.jbc.2026.113395

**Published:** 2026-08-05

**Authors:** Bryce Dye, Richard Dunn, Kathryn Wheeler, Grace Lee, Nicholas Pittner, Corina Maeder

**Affiliations:** Department of Chemistry, Trinity University, San Antonio, Texas, USA

**Keywords:** RNA splicing, spliceosome, mRNA, ribonuclear protein (RNP), protein stability, U5 snRNP, spliceosome assembly

## Abstract

The spliceosome catalyzes pre-messenger RNA splicing by facilitating two transesterification reactions that excise introns and ligate exons together. To accomplish this process, the spliceosome assembles and rearranges its components through a series of sequentially regulated steps. A key step in this pathway is the transition to the catalytically active B^act^ complex. During its catalytic activation, the spliceosome must release Dib1, an essential, conserved 143 amino acid protein that resides in the U4/U6-U5 tri-snRNP complex. However, the mechanism of Dib1 departure is not fully characterized. To further this understanding, this study determined the importance of the flexible C-terminus of Dib1 in the spliceosome. The C-terminus of Dib1 sits in proximity to the catalytic regions of both U5 and U6 snRNAs and interacts with Prp8 and Prp31 in the spliceosome. We find that the length of Dib1’s C-terminus is critical for the stability of the protein, and its removal results in loss of cell viability and splicing capabilities. Additionally, we have identified residues in Dib1’s C-terminal tail that we postulate are part of stabilizing protein–protein interactions in the spliceosome, including key areas between Dib1 and Prp8. Together, these findings further our understanding of a critical step in spliceosome assembly and activation.

Pre-messenger RNA (pre-mRNA) splicing is essential for eukaryotic gene expression, where constitutive and alternative splicing generates diverse mRNA transcripts that produce multiple protein isoforms from a single gene (1). During the splicing process, the intronic regions of pre-mRNA are excised while the remaining exonic regions are ligated together to produce a mature mRNA. This process is facilitated by the spliceosome, which catalyzes two transesterification reactions to accomplish pre-mRNA splicing. The spliceosome is a large, ribonucleoprotein complex composed of five small-nuclear RNAs (snRNAs), snRNA-associated proteins that bind to form small ribonucleoprotein complexes (snRNPs) and additional auxiliary proteins (2). The five snRNP components of the spliceosome are the U1, U2, U5, and the U4/U6 snRNPs. Individual snRNPs are formed before spliceosome association and undergo rearrangements during spliceosome assembly, catalysis, and dissociation (3, 4, 5). The snRNP components of the spliceosome are recruited to the pre-mRNA in distinct sequential steps to achieve proper catalysis (Fig. 1). The pre-mRNA-associated machinery moves through eight canonical conformations characterized by the association and dissociation of spliceosomal components to catalyze pre-mRNA splicing (2).

**Figure 1:**
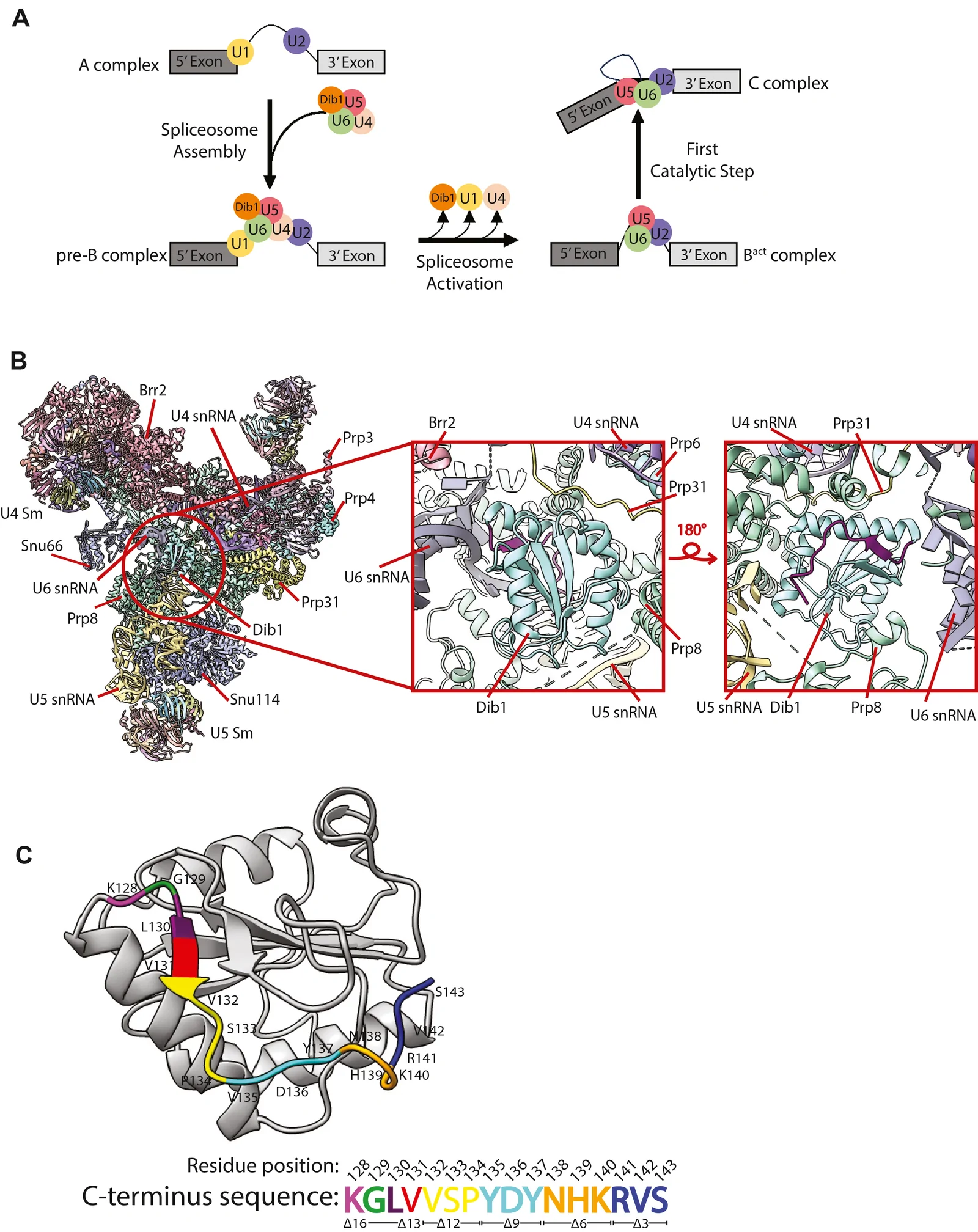
**Dib1 in thespliceosome and the Dib1structure.***A*, Schematic representation of spliceosome assembly and activation highlighting the incorporation and departure of Dib1. *B*, *S. cerevisiae* U4/U6–U5 tri-snRNP with the central location of Dib1 (*cyan*) and its C-terminal tail (*fuchsia*) shown. Inset shows ∼180° rotation to highlight the C-terminal tail of Dib1 (*fuchsia*) (PDB: 5GAN) (23). *C*, Structure of Dib1 showing the main truncation sites (PDB: 5NRL) (11). The C-terminal tail is colored to represent the number of residues truncated from the C-terminal tail end of Dib1 with the corresponding sequence shown.

The first step in spliceosome formation is the association of the U1 snRNA with the 5′ splice site (ss), followed by the binding of the U2 snRNP to the branch site (bs) to form the A complex (3). The U4/U6-U5 tri-snRNP is then recruited to the growing complex by the formation of a short duplex between the 5′ and 3′ ends of the U2 and U6 snRNAs, forming the pre-B complex (6). The B complex is subsequently formed by dissociation of the U1 snRNA from the 5′ splice site and binding of the U6 snRNA, which is mediated by the Prp28 helicase (6). The B complex is the final pre-catalytic state of the spliceosome. In the transition from the B complex to the B^act^ complex, the U4 snRNP and several U5-associated proteins are released from the spliceosome, while additional proteins are recruited to the spliceosome (2) (Fig. 1*A*).

Among the U5-associated proteins that depart in the catalytic transition is Dib1, a 17-kDa protein that resides in the catalytic core of the pre-catalytic spliceosome (Fig. 1*B*) and is essential for splicing and cell viability (7, 8). The Dib1 protein has been remarkably conserved throughout the evolution of distant species. Yeast Dib1 shares ∼63% sequence identity with the human homolog, hDim1. The structure of Dib1 closely resembles that of thioredoxin-like proteins with a C-terminal extension (Fig. 1*C*); however, Dib1 lacks oxidoreductase activity like other Dib1 family proteins (8). hDim1 has been shown to play a unique role in tri-snRNP assembly. hDim1 is tethered to the U5 snRNP by chaperone protein CD2BP2 (also known as U5–52K), which occupies the same binding site as hDim1 in the immature U5 snRNP (9). As the final associated proteins are recruited to the U5 snRNP, hDim1 is exchanged for CD2BP2 and CD2BP2 is released, which signals for the binding of the U5 snRNA with the U4/U6 di-snRNP (9). In the yeast U4/U6-U5 tri-snRNP, Dib1 forms stable intermolecular interactions with the N-terminal α-helices of Prp8 and a disordered region of Prp31 (2, 10). Both the U5 loop I and the U6 5′ ss sequence reside in close proximity to Dib1 and Prp8 (Fig. 1*B*) (2, 10). Cryo-EM data has shown that Dib1’s C-terminal tail specifically resides in close proximity to the U6 and U5 snRNAs, as well as Prp8, suggesting that this region may be important in stabilizing the necessary interactions facilitated by Dib1 (11). Given the flexible nature of this region and its lack of significant secondary structure, we are defining this region as the C-terminal tail (Fig. 1*C*). The importance of Dib1’s C-terminal tail is further suggested by its high degree of sequence conservation between species (Fig. S1). The conservation of the C-terminal tail suggests that this region may facilitate important interactions that have been maintained by evolution.

The departure of Dib1 from the B complex spliceosome is necessary for the catalytic activation of the complex (2). Due to the spatial proximity of Dib1 to the catalytic U5 and U6 snRNAs, the protein has been theorized to act as a linchpin in the catalytic core of the spliceosome by inhibiting catalytic activity until the catalytic core is occupied by the pre-mRNA (7). While the mechanism of Dib1 departure from the spliceosome is not fully understood, contributing factors to its role have been identified. Destabilizing mutations to Dib1 at L76/D78 or F85 induce a temperature-sensitive (*ts*) phenotype for cell growth and splicing activity (7), suggesting a role for critical Dib1 interactions in the core of the spliceosome. Furthermore, deletions to the hDim1 promoter region result in dysregulation of hDim1 expression and have been implicated in Burn-McKeown syndrome, a craniofacial developmental disorder (12). Finally, *in vitro* assays have shown that the C-terminal tail of hDim1 is flexible, likely aiding in the dynamic role of hDim1 within the human spliceosome (13). The flexible nature of the C-terminal tail may provide a means to transiently interrupt interactions between Dib1 and catalytic regions of the spliceosome as the complex activates (2).

Further investigation into the role of Dib1’s C-terminal tail is necessary as it has been identified as a region with unique flexibility and maintains important interactions in the pre-catalytic spliceosome (11, 13). In this study, we investigated the importance of the length and residue identity of the Dib1 tail region through a series of deletion and alanine substitution mutants. We have identified a minimum C-terminal tail length that is necessary for cell viability and pre-mRNA splicing functionality. Additionally, we find that the C-terminal tail of Dib1 is required for intramolecular stabilization, with the length of the tail relating to protein stability. We have also identified two potential residues in the C-terminal tail of Dib1 that support its function through a proposed hydrophobic interaction with Prp8. Together, these findings identify a multifaceted purpose for the C-terminal tail of Dib1 in the spliceosome and identify specific interactions that appear to be important for spliceosome activation.

## Results

### C-terminal of Dib1 is necessary for yeast growth

**Figure 2:**
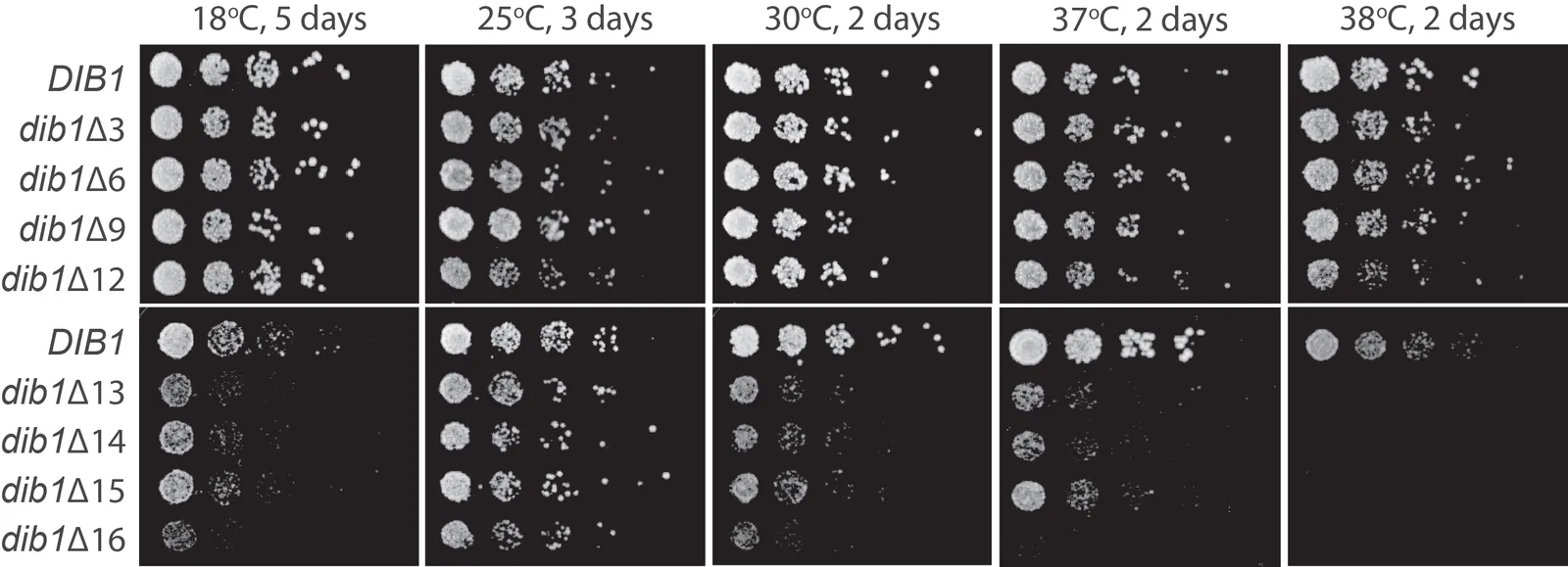
**Representative viability analysis for *S. cerevisiae dib1*Δ cells expressing wild-type (*DIB1*) or truncated versions (*dib1Δ*C) as the sole copy of the *dib1* gene.** Serial dilutions were spotted onto rich medium (YPD) and grown at varying temperatures for indicated numbers of days. Viability assays were performed with three independent biological replicates.

Cryo-EM structures of spliceosomal complexes have placed Dib1’s C-terminal tail in proximity to Prp8 and the U5 and U6 snRNAs, suggesting an important role for the tail region (Fig. 1*B*) (10, 11). To explore the role of Dib1’s C-terminus in the spliceosome, we created a series of *Saccharomyces cerevisiae* strains harboring C-terminal truncations of *dib1* as the sole copy of the dib1 gene. *DIB1* is essential for viability in all eukaryotes. C-terminal truncations removed up to 16 residues in one or three-amino-acid increments (Fig. 1*C*). Serial dilution growth assays were performed to assess whether the C-terminal deletions impacted yeast growth rate. Five temperatures, 18, 25, 30, 37 and 38 °C, were surveyed in the growth assays to explore the effects of temperature on yeast growth rate (Fig. 2).

In examining the growth assays, removal of up to 12 residues was well tolerated by the yeast strains at all temperatures (Fig. 2). Only *dib1Δ12* showed a slight temperature sensitivity at 38 °C in comparison to wild type. Removal of additional residues resulted in severe temperature-dependent growth defects. Deletion of residues 13 to 16 resulted in slowed or severely affected growth at all temperatures, including the permissive temperatures. The most severe was the truncation to residue 16, which resulted in cell lethality at 37 and 38 °C with minimal detection of growth at 18 °C and 30 °C. Together, these assays identify that residues 13 to 16 are critical for the function of Dib1.

### C-terminal tail of Dib1 is required for both intramolecular stabilization and intermolecular interactions

The observed temperature sensitivity of the yeast truncation strains can be explained in two ways. First, the Dib1 protein itself is unable to fold into a functional, stable protein without the tail region. Alternatively, the tail residues are critical for intermolecular interactions with the neighboring spliceosomal components. To differentiate between these two rationales, we determined the ΔG_unfolding_ for the truncated Dib1 proteins using denaturing circular dichroism spectroscopy to investigate how C-terminal deletions impact Dib1 stability.

**Figure 3:**
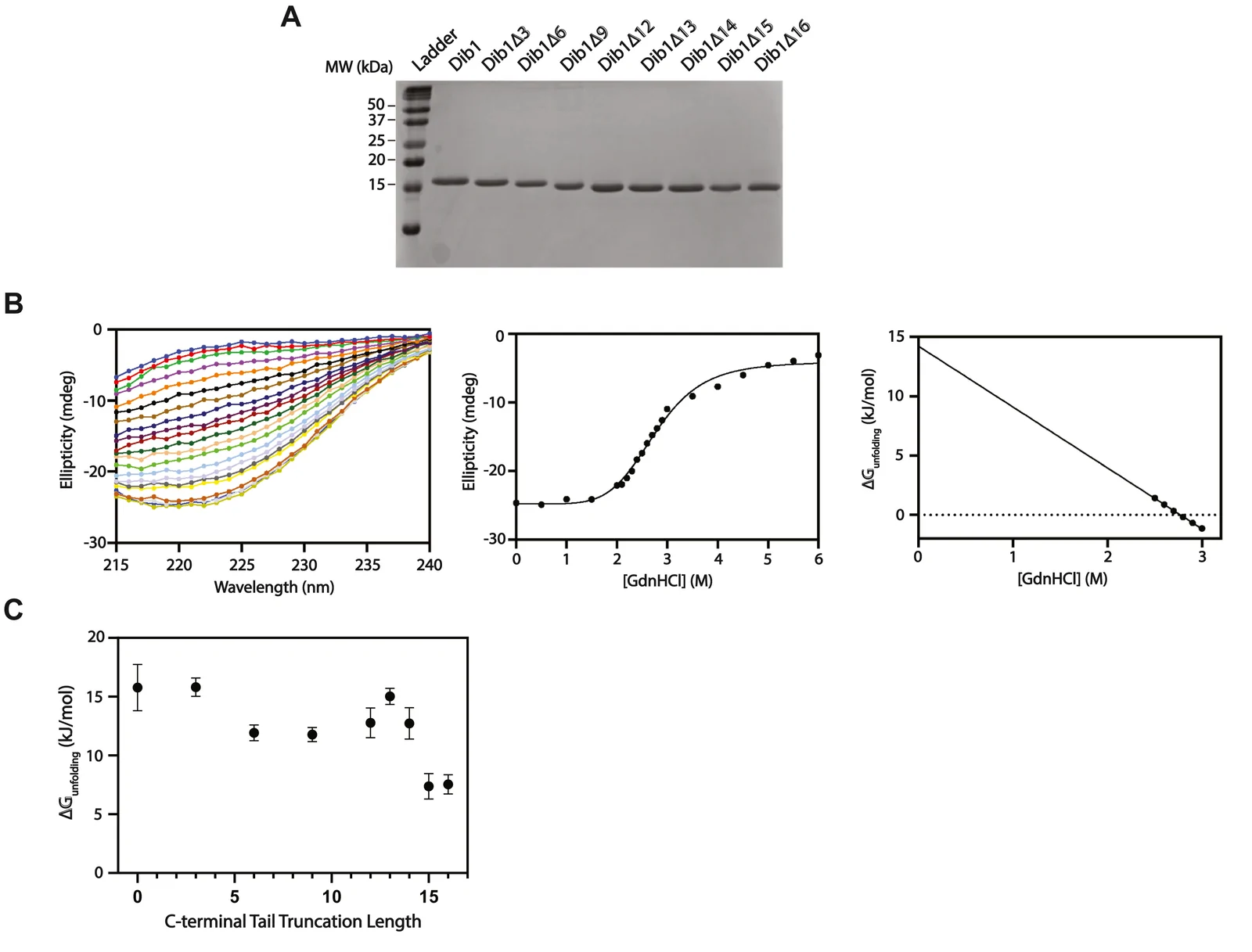
**Purification anddenaturationstudies of Dib1 proteins.***A*, SDS-PAGE of purified Dib1 and Dib1Δ truncation proteins yielded a single band when stained with Coomassie brilliant blue. A decrease in the molecular weight of Dib1Δ proteins is visible relative to full-length Dib1. *B*, representative Chemical Denaturation Experiment. *Left*. Far-UV CD spectra of 9 μM full-length Dib1 titrated with increasing concentrations of GdnHCl. *Middle*. Chemical unfolding of Dib1 monitored by the ellipticity at 220 nm. *Right*. The ΔG_unfolding_ for full-length Dib1 was plotted as a function of GdnHCl concentration. *C*, ΔG_unfolding_ for the Dib1 protein *versus* the deletion length of its C-terminal tail with increasing deletion lengths in increments of one or three amino acids. Significant destabilization of the protein occurs as six and fifteen residues are truncated from Dib1’s tail. With the exception of Dib1Δ13, Dib1 can maintain its stability at ∼13 kJ/mol through removal of six to fourteen residues from its tail.

For denaturing circular dichroism, purified Dib1 protein samples (Figs. 3*A* and S2) were incubated with increasing concentrations of guanidinium hydrochloride ([GdnHCl]). The ellipticity of the sample was then measured in the range of 195 to 240 nm. Overlay spectra showed a characteristic unfolding of the protein as additional denaturant was added. Ellipticity at 220 nm was plotted against the [GdnHCl] to produce a sigmoidal denaturation curve. ΔG_unfolding_ was extracted from this curve using a two-state analysis (Figs. 3*B* and S3–S5). These findings are summarized in Table 1 and Figure 3*C*. Deletion of six residues (Dib1Δ6) resulted in a stability of 11.9 kJ/mol, which is a significant decrease of ∼4 kJ/mol in stability compared to the wild-type Dib1. The protein stability did not decrease below ∼12 kJ/mol until removal of 15 C-terminal residues (Fig. 3*C*). Given the lack of growth defect for C-terminal truncations through 12 residues, the yeast appeared to tolerate the initial loss of stability in Dib1. Interestingly, deletion of residue 13 showed a slight stabilization relative to smaller truncations despite significant growth defects not observed in *dib1Δ12* mutants (Fig. 2). Further deletion of residue 14 resulted in proteins with identical stability to Dib1Δ12, but cells expressing *dib1Δ14* still exhibited significant growth defects. These findings suggest an intermolecular role for these residues (see below). Deletions of residues 15 and 16 resulted in further destabilization with a ΔG_unfolding_ of ∼7.5 kJ/mol, constituting a ∼53% reduction in intramolecular stability from wild type Dib1. These final two truncations were also associated with a decrease in the *m*-value of the ΔG extrapolation plot (Table S1). The *m-*value is significantly correlated to the change in solvent accessible surface area of a protein upon denaturation (14). Thus, Dib1Δ15 to 16 may not be stabilized into a fully folded state as the change in solvent accessibility between its native and denatured states is much smaller than observed in other Dib1 truncations.

**Table 1:** Stability values of Dib1 and Dib1Δ mutants

Protein	Molecular weight (amu)	Phenotype at 37 °C	ΔG_u_° (kJ/mol)
Dib1	17,057	*wt*	15.8 ± 2.0
Dib1Δ3	16,715	*wt*	15.8 ± 0.8
Dib1Δ6	16,335	*wt*	11.9 ± 0.7
Dib1Δ9	15,894	*wt*	11.8 ± 0.6
Dib1Δ12	15,610	*wt*	12.8 ± 1.3
Dib1Δ13	15,511	*ts*	15.0 ± 0.7
Dib1Δ14	15,398	*ts*	12.7 ± 1.3
Dib1Δ15	15,341	*ts*	7.4 ± 1.1
Dib1Δ16	15,213	*ts*	7.5 ± 0.8
Dib1Δ14 G129A	15,413	*ts*	13.2 ± 1.0
Dib1Δ15 K128A	15,284	*ts*	16.0 ± 1.0

### Splicing defects correlate with yeast growth

**Figure 4:**
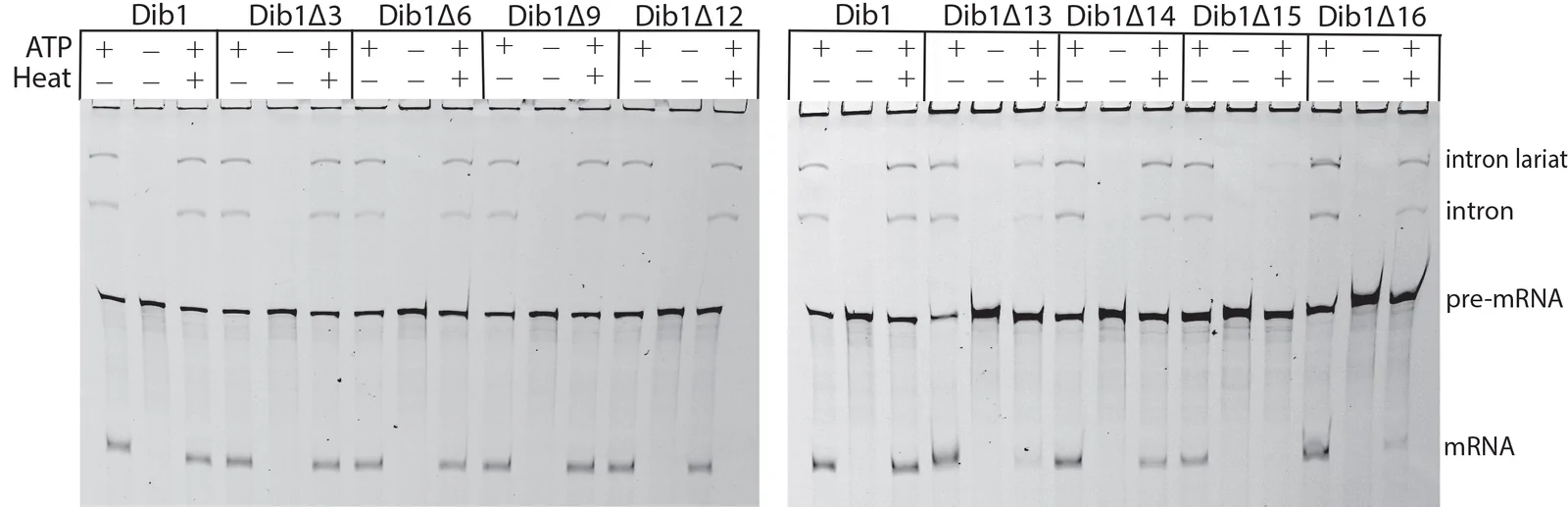
**Pre-mRNA splicing of fluorescently labeled actin pre-mRNA transcript.** Conditions in the assay are indicated. For samples with ATP, ATP was added to a final concentration of 10 mM. Non-ATP samples were treated with glucose to deplete ATP. Splicing reactions were allowed to proceed for 30 min at 25 °C and then visualized by denaturing PAGE using fluorescence at 650 nm. Heat-treated extracts were incubated at 38 °C for 18 min followed by 5 min on ice and 15 min at 25 °C prior to the reaction.

Having assessed the effect of Dib1’s C-terminal tail length on protein stability and yeast growth, we next determined how C-terminal truncations impacted pre-mRNA splicing using *in vitro* splicing assays. Cellular splicing extracts were made from yeast harboring *dib1* truncations as previously described (7). Cy5-labeled ACT1 pre-mRNA was used to monitor ATP-dependent splicing in the cellular extracts. Denaturing polyacrylamide gels were used to visualize splicing products. For each extract, three reactions were performed: one negative control reaction in the presence of glucose and no ATP addition, one positive control reaction with non-heat-shocked extracts and ATP addition, and one experimental reaction with both heat-shocked extracts and ATP addition (Fig. 4). Glucose addition was used in the absence of ATP to aid ATP depletion. Heat shock of extracts at 38 °C mimicked growth conditions in the viability assays. All experiments were conducted with triplicate biological replicates and were consistently reproducible.

The level of splicing observed in the presence of full-length Dib1 was similar to that observed in extracts with Dib1 tail deletions of up to twelve residues (Fig. 4). Dib1Δ12 exhibited a reproducible, slightly reduced splicing activity upon heat-shock treatment but reactivity was still observed to proceed at an appreciable amount. The removal of additional residues from Dib1’s C-terminus resulted in a precipitous loss of splicing activity upon heat-shock (Fig. 4). This finding was consistent with the results of the growth assays which showed a minute temperature sensitivity with *Δ*12 mutants and dramatic growth impairment for additional truncations (Fig. 2). DibΔ13 showed a significant loss of splicing activity, while Dib1Δ14 was not reduced to the same extent. It was also observed that, upon removal of 15 amino acids from Dib1’s tail, spliceosome activity was completely inhibited when heat-shocked. Activity of this truncation aligns with the previously observed large drop in the protein stability of Dib1 (Fig. 3*C*), suggesting intramolecular Dib1 stability may be related to spliceosomal activity. Even still, Dib1Δ15 and Dib1Δ16 maintain some level of growth and splicing at the permissive temperature. While splicing extracts inherently have some variability, the phenotypic trends that were observed were consistent over multiple extract preparations and splicing assays.

### Residues L130 and V131 facilitate intermolecular interactions with Dib1’s C-terminal tail

**Figure 5:**
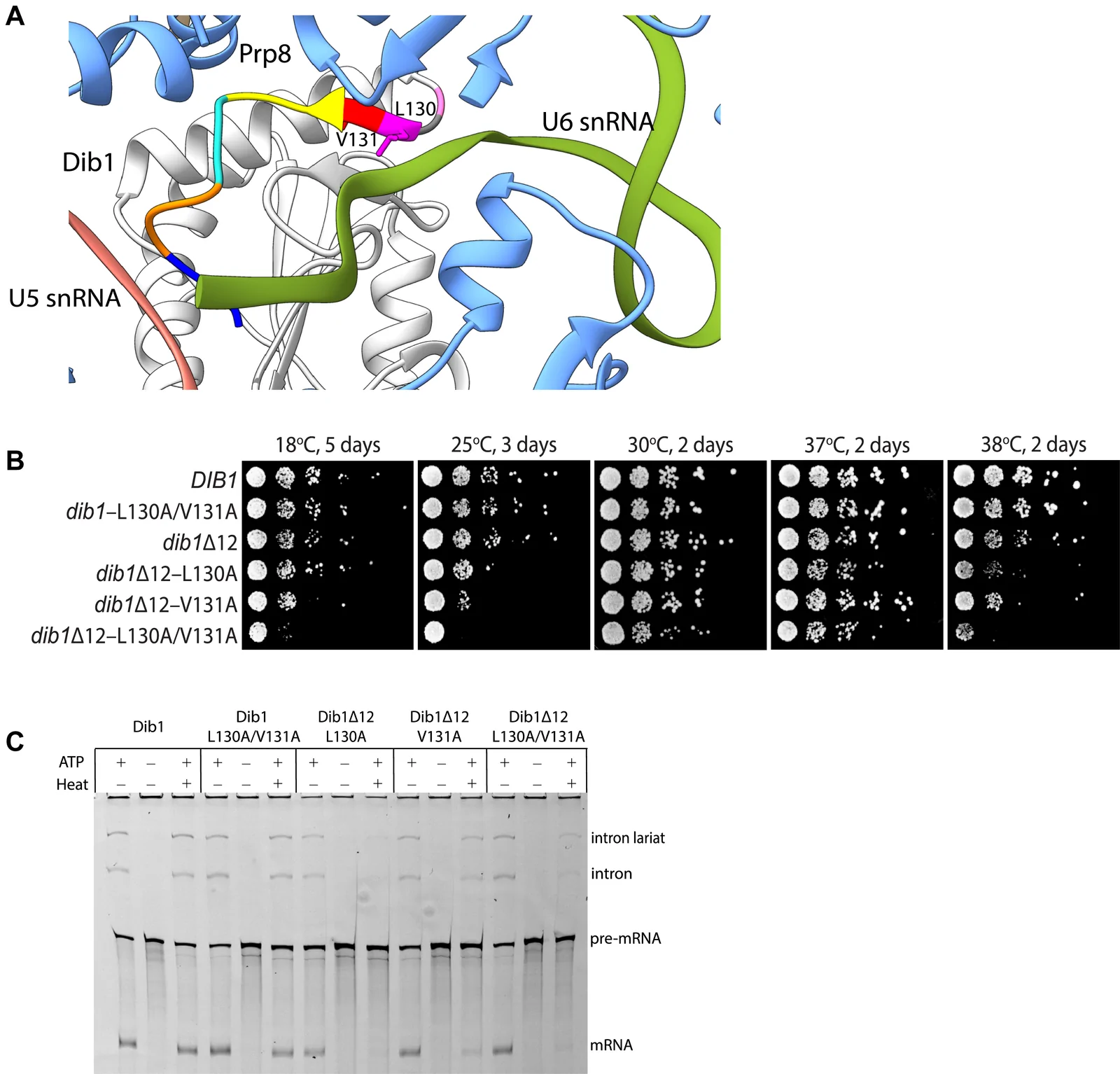
**Impacts of L130 and V131 in the C-terminal tail of Dib1.***A*, Interactions of the C-terminal tail of Dib1 in the context of the pre-catalytic spliceosome (PDB: 5NRL) (11). The C-terminal tail of Dib1 (*light grey*) is colored based on the truncation length with L130 (*magenta*) and V131 (*red*) highlighted. U6 snRNA (*green*), Prp8 (*light blue*) and U5 snRNA (*orange*) are shown, with the region of Prp8 that lies in front of Dib1 omitted for better visibility of Dib1. *B*, viability analysis for *S. cerevisiae dib1*Δ cells expressing wild-type (*DIB1*), full-length Dib1 with L130A/V131A, or the Dib1Δ12 version with or without alanine mutations is indicated in the figure. *C*, pre-mRNA splicing of fluorescently-labeled actin pre-mRNA transcript with extract harboring Dib1 and Dib1Δ12 with L130A and V131A mutations. Conditions in the assay are as indicated. For samples with ATP, ATP was added to a final concentration of 10 mM. Non-ATP samples were treated with glucose. Splicing reactions were allowed to proceed for 30 min at 25 °C and then visualized by denaturing PAGE using fluorescence at 650 nm. Heat-treated extract were incubated at 38 °C for 18 min followed by 5 min on ice and 15 min at 25 °C prior to the reaction.

The ΔG_unfolding_ of Dib1Δ13 and Dib1Δ14 did not display any notable decreases relative to mutants Dib1Δ6 to Dib1Δ12, but truncations of these later residues still influenced growth and splicing. These findings suggest a potential intermolecular role for these residues which lie in close proximity to the U6 snRNA and Prp8 (Fig. 5*A*). To investigate these roles, alanine substitutions were incorporated at residues L130 and V131 in both full-length and truncated Dib1 (Dib1Δ12). L130 and V131 are conserved from yeast to human (Fig. S1). The impacts of alanine substitution on cell growth and viability were assessed using yeast growth assays, as previously performed. Full-length Dib1 expressing both alanine substitutions did not exhibit growth defects (Fig. 5*B*). However, Dib1*Δ*12 with L130A was both cold and heat sensitive, while DibΔ12 with V131A was only cold sensitive. Incorporation of both L130A and V131A in Dib1Δ12 showed temperature sensitivity at cold and elevated temperatures (Fig. 5*B*). This finding suggests that the identity of these residues in the C-terminal tail of Dib1 contributes to the function of the Dib1 protein, which may explain why the removal of these residues in Dib1Δ13 and Dib1Δ14 showed unanticipated growth defects despite not impacting their intramolecular stability.

To confirm that the observed temperature-sensitive growth of Dib1Δ12 L130A and V131A mutants was due to splicing catalysis defects, *in vitro* splicing was surveyed for yeast extracts incorporating these mutants as their sole copy of Dib1 (Fig. 5*C*). Consistent with the growth assays, full-length Dib1 mutated with L130A/V131A exhibited a reproducible, slightly reduced loss of splicing when heat-shocked. However, splicing extracts consisting of Dib1*Δ*12 with either L130A or V131A displayed a dramatic decrease in splicing catalysis after heat-shock, with Dib1Δ12-L130A showing a more severe phenotype (Fig. 5, *B* and *C*). Furthermore, extracts possessing Dib1*Δ*12-L130A/V131A also yielded minimal splicing after heat shock, which is consistent with the observed growth defects. This splicing defect is in contrast to Dib1Δ12 which maintained the ability to splice at non-permissive temperatures (Fig. 4). These protein variants could not be purified after several different purification trials, thus, we were unable to compare how these mutations affect the stability of the protein.

### Increasing protein stability does not recover splicing activity in truncated Dib1 samples

Given the finding that the identities of the 13th and 14th residues of the C-terminal tail contribute to the function of Dib1, we sought to determine if the stability defects of Dib1Δ15 and Dib1Δ16 alone accounted for their further loss of function in growth and splicing assays. To investigate the importance of these two residues, alanine substitutions were made to the terminal residue in Dib1Δ14 and Dib1Δ15 yielding Dib1Δ14-G129A and Dib1Δ15-K128A. As before, these mutants were examined using protein stability, yeast growth assays and *in vitro* splicing assays. While no change to protein stability was observed for Dib1Δ14-G129A relative to Dib1Δ14, we did observe a significant stabilization from the Dib1Δ15-K128A mutation from 7.4 kJ/mol to 16.0 kJ/mol, which is equivalent to the stability of full-length Dib1 (Table 1).

**Figure 6:**
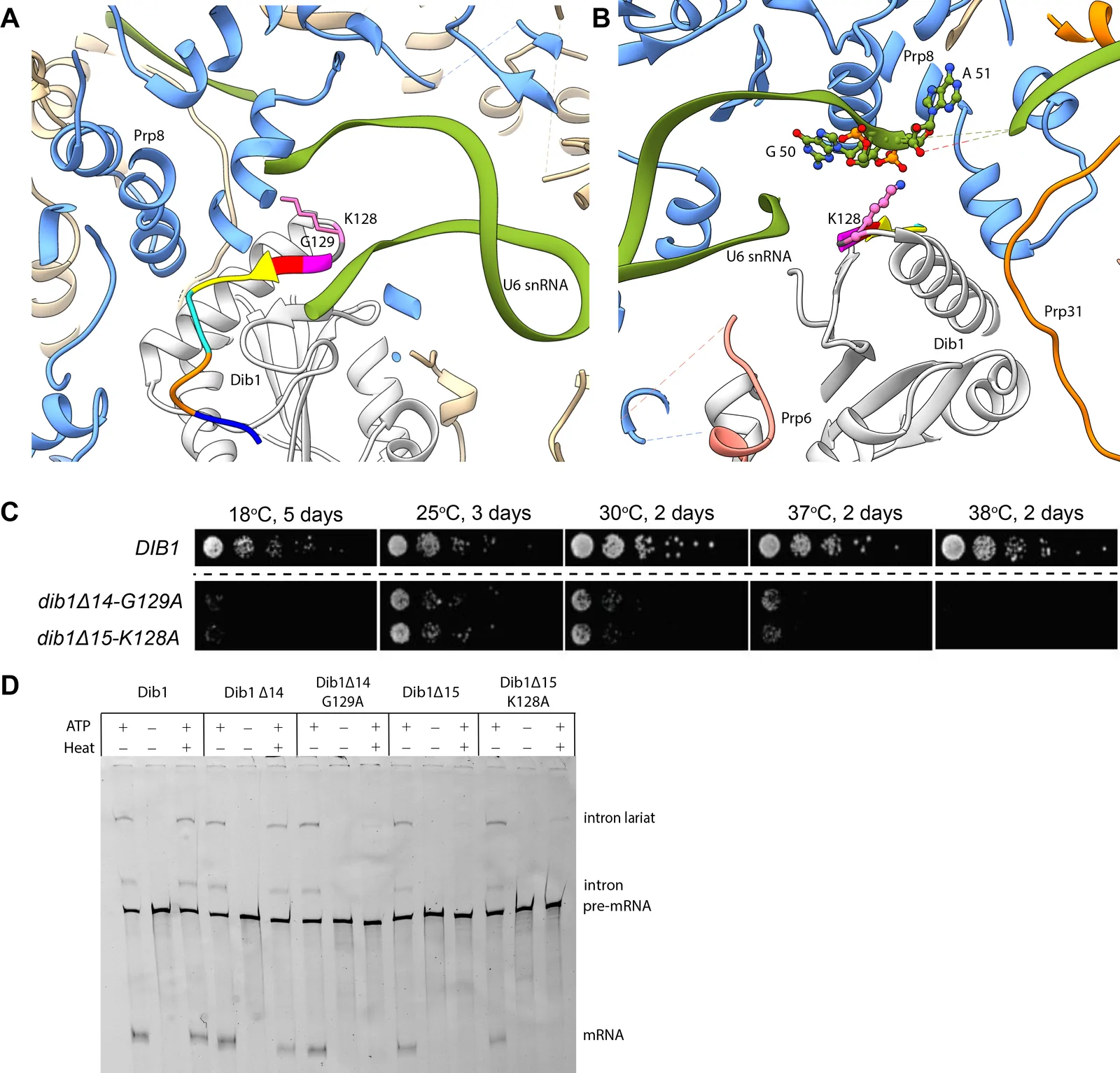
**Impacts of G129 and K128 in the C-terminal tail of Dib1.***A*, interactions of the C-terminal tail of Dib1 in the context of the pre-catalytic spliceosome (PDB: 5NRL) (11). The C-terminal tail of Dib1 (*light grey*) is colored based on the truncation length with K128 (*pink*) and G129 (*dark grey*) highlighted. U6 snRNA (*green*) and Prp8 (*light blue*) are shown, with the region of Prp8 that lies in front of Dib1 omitted for better visibility of Dib1. *B*, *ball* and *stick* representation of interactions of the C-terminal tail of Dib1 in the context of the pre-catalytic spliceosome (PDB: 5NRL) (11). The C-terminal tail of Dib1 (*light grey*) is colored based on the truncation length with K128 (*pink*) highlighted. U5 snRNA (*orange*), Prp8 (*light blue*) and U6 snRNA (*green*) with G50 and A51 represented as *ball* and *stick* are shown. *C*, viability analysis for *S. cerevisiae dib1*Δ cells expressing wild type (*DIB1*), Dib1Δ14-G129A or Dib1Δ15-K128A as the sole copy of the *dib1* gene, as indicated in the figure. *(D)* Pre-mRNA splicing of fluorescently-labeled actin pre-mRNA transcript with extract harboring Dib1 and Dib1Δ14 with or without G129A and Dib1Δ15 with and without K128A mutations. Conditions in the assay are as indicated. For samples with ATP, ATP was added to a final concentration of 10 mM. Non-ATP samples were treated with glucose. Splicing reactions were allowed to proceed for 30 min at 25 °C and then visualized by denaturing PAGE using fluorescence at 650 nm. Heat-treated extract were incubated at 38 °C for 18 min followed by 5 min on ice and 15 min at 25 °C prior to the reaction.

To investigate if stabilization of the Dib1Δ15 protein would rescue cell growth and splicing activity, yeast growth assays were performed with strains expressing Dib1Δ14-G129A and Dib1Δ15-K128A. We observed temperature-sensitive growth for both Dib1Δ14-G129A and Dib1Δ15-K128A (Fig. 6*C*), suggesting that despite re-stabilization of the Dib1Δ15-K128A protein, function was not rescued. Furthermore, heat-shocked splicing extracts containing Dib1Δ14-G129A and Dib1Δ15-K128A both showed minimal splicing activity (Fig. 6*D*). These observations suggest that, while the stability of Dib1 has been shown to be important, there are other factors within Dib1 that impact spliceosomal efficacy. The reported defects may not simply be a result of protein destabilization but also potentially a loss of intermolecular interactions, as these residues lie in close proximity to U6 snRNA and Prp8 (Fig. 6*A*).

## Discussion

The spliceosome, as a macromolecular enzymatic complex, performs its catalysis by moving through several conformational rearrangements. These rearrangements are characterized by the remodeling of intermolecular interactions between its protein and snRNA components (15). The accuracy of these sequential rearrangements through the splicing cycle is necessary for pre-mRNA splicing fidelity and appropriate substrate recognition (15, 16, 17). In a key rearrangement, Dib1, which resides in the catalytic core of the pre-catalytic B splicing complex, departs from the spliceosome as the complex transitions to its catalytic state (11, 18, 19). In this transition to the B^act^ complex, the U4 snRNP and several U5-associated proteins, including Dib1, are released from the spliceosome, while additional proteins are recruited to the spliceosome (2). This catalytic activation is driven by the unwinding of the U4/U6 duplex by DEAH-box helicase Brr2, later forming catalytic B^∗^ complex through further action of the Prp2 helicase (11).

In this work, we have characterized the flexible C-terminal tail of the Dib1 protein and its role in the splicing cycle. Truncation studies revealed that Dib1’s C-terminal tail is essential for both intramolecular stabilization (Table 1 and Fig. 3*C*) and splicing efficacy (Fig. 4) independently. Temperature-sensitive phenotypes resulting from mutations to biological complexes commonly arise from either destabilization of the mutated molecule, or a loss of affinity for binding interactions which are exposed by the elevated temperature (20). From our findings, it is possible that we are observing both types of defects. In the case of the C-terminal tail of Dib1, temperature-sensitive phenotypes and significant loss of intramolecular stability occur at separate and discrete truncations (Δ13 and Δ15, respectively). Therefore, the temperature-sensitive cell growth and splicing patterns (Figs. 2 and 4) may be due to a combination of intramolecular destabilization and loss of intermolecular interactions with surrounding splicing machinery. Previous work has shown that the function of yeast Dib1 can be rescued by expression of human hDim1; however, expression of hDim1Δ14 results in a temperature-sensitive phenotype, indicating that the C-terminal tail is important for function (21). In addition to the phenotypes observed with the C-terminal truncations, several temperature-sensitive Dib1 mutants have been characterized by a loss of intramolecular stability, indicating the importance of Dib1 stability to splicing efficacy (7).

**Figure 7:**
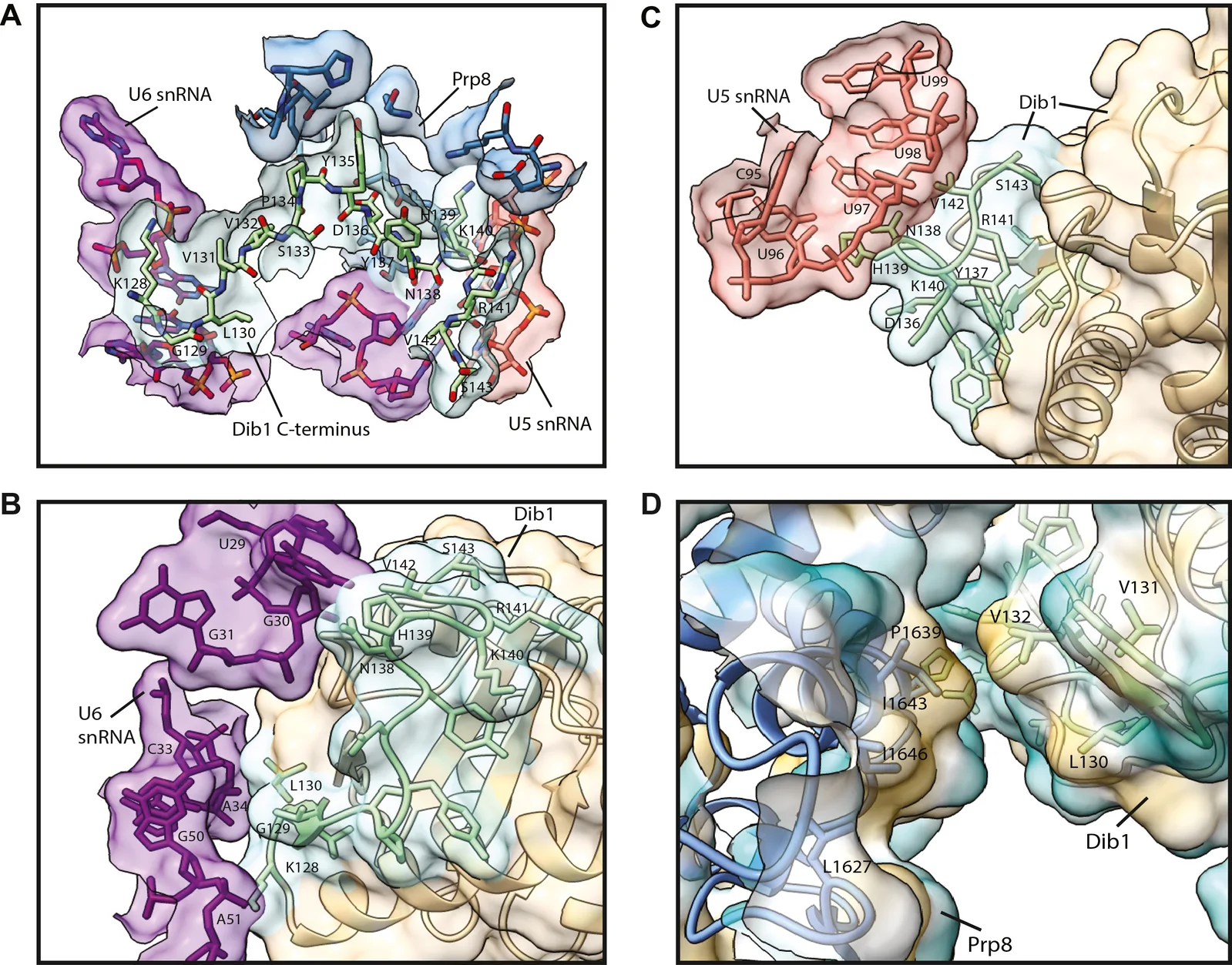
**Interactions of the C-terminal tail of Dib1 withkeysplicingcomponents.***A*, Zoomed-in atomic and surface view of Dib1’s C-terminus (*pale green*) and its interactions with the U5 snRNA (*red*), U6 snRNA (*purple*), and Prp8 (*blue*) (PDB: 5NRL) (11). Residues of Dib1’s C-terminus are labeled with identity and position. *B*, ribbon diagram and surface depiction of the interface between Dib1’s C-terminal tail (Dib1 in wheat with the C-terminal tail in *pale green*) and the U6 snRNA (*purple*). Dib1’s C-terminus is in close-contact with the phosphate backbone of the U6 snRNA and with several ribonucleotides. *C*, ribbon diagram and surface depiction of the interface between Dib1’s C-terminal tail (Dib1 in wheat with the C-terminal tail in *pale green*) and the U5 snRNA (*red*). Residues of Dib1’s C-terminus are in close-contact with the phosphate backbone of the U5 snRNA. *D*, ribbon diagram and surface depiction of the interface between the C-terminal tail of Dib1 (*pale green*) and Prp8 (*blue*) zoomed in to display specific interactions with L130 and V131 of Dib1. The surface hydrophobicity is demonstrated with more hydrophobic regions in *yellow* and more hydrophilic regions in *blue*. Dib1’s C-terminal tail is in close-contact with several residues of Prp8. L130 and V131 may form essential interactions with hydrophobic residues of Prp8 that are required for stabilization of the splicing complex.

Structures of the B complex have revealed that Dib1’s C-terminal tail interacts with the U5 and U6 snRNAs as shown in Figure 7*A* (11). The ε-amino group of K128 in Dib1 is positioned ∼2.6 Å from the phosphodiester bond between G50 and A51 of the U6 snRNA and is appropriately oriented to form electrostatic or hydrogen-bonding interactions. Similarly, the carboxamide group of N138 is ∼3.5 Å away from the 2′-hydroxyl of G30 in U6, and the amide nitrogen in the backbone of G129 is ∼3.2 Å away from the 2′-hydroxyl of A34 in U6. Both are positioned appropriately to form hydrogen bonds between the C-terminus of Dib1 and the U6 snRNA (Fig. 7*B*) (11). The C-terminus of Dib1 is also in close proximity and correctly oriented to form stabilizing interactions with the U5 snRNA. This includes the backbone carbonyl of H139 and the backbone carbonyl of R141 in Dib1, which are ∼3.2 Å and ∼3.5 Å, respectively, away from the 2′-hydroxyl group in U97 of the U5 snRNA (Fig. 7*C*). While these structures provide a static image along a dynamic pathway, these observations highlight the importance of C-terminal tail of Dib1 in the active site of the spliceosome.

In addition to Dib1’s C-terminal tail interactions with the U5 and U6 snRNA, the C-terminal tail of Dib1 appears to interact with Prp8 (11). Residues V131 and L130 on Dib1’s C-terminal tail (corresponding to the terminal residues in Dib1Δ12 and Dib1Δ13) reside in an exterior hydrophobic patch that appears to interact with the hydrophobic residues P1639, I1643 and I1646 in Prp8 (Fig. 7*D*) (11). These hydrophobic residues in Prp8 reside within the C-terminal Endonuclease-like domain and linker regions of Prp8, which form the spliceosomal active site cavity and coordinate the U5 and U6 snRNAs (22, 23). While we have identified key residues in this structure, this region is expected to be quite dynamic during the splicing cycle. Nearby residues in this area of Prp8 were identified in screens for suppressors for U4-cs1, a cold-sensitive U4 snRNA that stalls spliceosome activation (24, 25). U4-cs1 is thought to inhibit spliceosome activation by preventing the ACAGA-box in U6 snRNA from interaction with the 5′ splice site. We found that deletion or alanine substitution of the largely hydrophobic, bulky V131 and L130 residues in Dib1, paired with the twelve-residue truncation mutant which includes the deletion of hydrophobic V132 in this same hydrophobic patch, induced temperature-sensitivity (Fig. 5). Based on our observations, the hydrophobic interactions between Dib1 and Prp8 in the B complex of the spliceosome are weakened *via* removal or substitution of L130, V131 and V132 to smaller residues, which led to destabilization of the overall pre-catalytic spliceosome. Given the conservation of L130 and V131, this hydrophobic patch and its potential interactions appear to be an important part of stabilizing this complex. These findings may be a contributing reason why this area arose in the U4-cs1 suppressor screen (24, 25). One important possibility is that this reduction in interactions, paired with the destabilization from the truncation, caused the splicing machinery to be more susceptible to conformational rearrangements or denaturation upon heat shock, resulting in the observed temperature-sensitive phenotype. These interactions may contribute to the stability, and the interaction of Dib1’s C-terminal tail with key splicing components highlights its potential role in stabilizing the B complex conformation (11). However, Dib1 interactions are transient, so these interactions must be overcome when Dib1 departs from the complex during the transition to the catalytically active spliceosome (16, 26).

The extent to which Dib1 intramolecular stability impacts pre-mRNA splicing has remained unclear (7). Dib1’s C-terminus was determined to be tightly related to the stability of the protein, as well as the ability to splice. However, the specific truncations that resulted in reduced splicing activity and a steep decline in intramolecular stability were uncoupled. The tolerance of the truncation of residues 130 to 138 without a change in stability may be indicative of the role of the flexible C-terminal tail in facilitating interactions between other molecules, rather than maintaining the integrity of Dib1. This would be supported by the observed temperature-sensitive phenotype without a decrease in protein stability when residues 130 to 131 are truncated (Figs. 2 and 3*C*.) The proposed interaction between Dib1 and Prp8 would explain these findings. Truncations beyond G129 in Dib1 result in a dramatic destabilization of the protein (Fig. 3*B*). The first truncation beyond this point (Dib1Δ15) corresponded with no visible splicing for heat-shocked splicing extracts (Fig. 4). The significant loss of splicing activity when the Dib1 protein is destabilized suggests that the intramolecular stability of Dib1, modulated by its C-terminal tail, may be an important factor in the spliceosome structure. This finding contrasts with the loss of splicing observed in the Dib1Δ13 mutation (Fig. 4), which may be related to intermolecular interactions in addition to stability.

One surprising finding was that mutating lysine 128 to an alanine in the context of the Dib1Δ15 mutation resulted in re-stabilization of the protein (Table 1), suggesting that this mutant could rescue the protein folding. However, expression of the stabilized Dib1Δ15-K128A mutant did not rescue yeast cell growth and exhibited both cold and heat sensitivity (Fig. 6, *C* and *D*). Additionally, splicing assays with Dib1Δ15-K128A extracts displayed no visible splicing product formation after heat-shock (Fig. 6*D*). This observation may be due to a loss of the possible electrostatic interactions between K128 and the phosphate backbone of the U6 snRNA (Fig. 6*B*), which could not be maintained by the alanine (7, 11). Residue 128 consistently has either a conserved lysine or arginine, highlighting the importance of a basic residue at that location (Fig. S1). Regardless, this finding showed that despite recovering stability equivalent to that of full-length Dib1, cell growth and splicing activity could not recover, which highlights the importance of the proposed intermolecular interactions facilitated by Dib1’s C-terminal tail. Overall, these studies clarify the importance of intermolecular interactions with Dib1’s C-terminal tail and surrounding splicing machinery for spliceosomal function. This builds on our previous work that identified stabilization of the spliceosome as a key component of the role for Dib1 (7).

In summary, we have demonstrated the importance of Dib1’s flexible C-terminal tail in spliceosome catalysis of pre-mRNA splicing. Truncation of Dib1’s C-terminal tail was observed to result in temperature-sensitive growth phenotypes and defects to splicing, as well as destabilization of the Dib1 protein. These findings hypothesize that the C-terminal tail is critical for Dib1 protein stability as well as key intermolecular interactions in the spliceosome. Additionally, substitution mutations performed in tandem with truncations revealed that the identities of conserved residues V131 and L130 are potentially important for maintaining intermolecular interactions between Dib1 and Prp8. The specific interaction facilitated by V131 and L130 with the addition of further deletion of the C-terminal tail appear to be important for stabilizing the B complex and allowing for proper coordination of the splicing machinery.

## Experimental procedures

### *S. cerevisiae* expression plasmids and strain creation

As previously described, *S. cerevisiae* expression plasmids were constructed for the *DIB1* gene ± 1 kb in pSE360 and pSE362 plasmids (7). pSE360 has a *URA3* gene, while pSE362 contains a *HIS3* marker. *S. cerevisiae dib1Δ* strain, with its endogenous *DIB1* gene knocked out, was transformed with the pSE360-*DIB1* plasmid (7). Truncation and alanine-substituted *dib1* mutant strains were made in *pSE362-DIB1* using PCR site-directed mutagenesis. Sequences for all primers are listed in Table S1. All mutants were confirmed by sequencing by Quintara Bio (Berkeley, CA). Mutant plasmids were incorporated into *S. cerevisiae dib1Δ* strains with pSE360-*DIB1* using traditional plasmid swapping techniques. Briefly, plasmids were transformed into the yeast and selected for on SD-His plates. These strains were then passaged on SD plates containing 5-FOA to confirm loss of the pSE360-*DIB1* plasmid. Incorporation of *pSE362-dib1* into the *S. cerevisiae dib1Δ* strain was confirmed with growth on SD-His and lethality on SD-Ura. Plasmids from these yeast strains were isolated and sequenced to confirm that the strains possessed the *dib1* mutants with the desired truncation.

### Recombinant protein expression plasmids

A previously created pET15b-*DIB1* bacterial expression vector was used to produce recombinant Dib1 protein (7). Truncation and alanine substitution mutants of the Dib1 protein were produced *via* PCR-based site-directed mutagenesis of the expression vector. PCR reactions were performed using iProof High Fidelity Polymerase (Bio-Rad). The PCR product was digested with Dpn1 for 4 to 8 hours and transformed into NEB-10 competent *E. coli* cells. Successfully transformed cells were plated on LB (Luria-Bertani medium) with ampicillin (100 μg/ml) plates at 37 °C. Transformants were cultured and isolated using the QIAgen mini-prep kit. Isolated plasmids were sequenced by Quintara Biosciences (Berkeley, CA).

### *S. cerevisiae* growth assay

*S. cerevisiae* colonies harboring pSE362-*dib1* plasmids were inoculated in YPD (yeast extract, peptone, dextrose medium) and grown overnight at 30 °C and 250 RPM. The overnight cultures were measured and diluted to an OD_600_ of 0.2. Diluted cultures were grown at 25 °C and 250 RPM until an OD_600_ of ∼0.4 was reached. Cultures were diluted again to an OD_600_ of 0.2, and a 1:6 serial dilution was created for each culture. A sterile multi-well pinner was used to transfer the cells to YPD plates. Plates were incubated to grow at 18, 25, 30, 37, and 38 °C for 2 to 5 days before imaging.

### *S. cerevisiae* spliceosome extraction and *in vitro* splicing

Whole cell yeast extracts were prepared as described previously (7). Splicing extracts were prepared in 20 mM HEPES (pH 7.9), 0.2 mM EDTA, 50 mM KCl, 20% glycerol, 0.5 mM DTT, 0.2 mM PMSF, and 0.5 mM benzamidine (7, 27, 28). Extracts were aliquoted and snap-frozen in single-use aliquots and stored at −80 °C.

The ACT1Δ6 plasmid was transformed into NEB-10 competent *E. coli* cells and purified with QIAprep Maxiprep Kit (Qiagen) (29, 30). The ACT1Δ6 plasmid was linearized with HindIII (New England Biolabs). Ethanol precipitation was performed to concentrate the linearized DNA. ACT1Δ6 pre-mRNA transcripts were synthesized *in vitro* by a T7 runoff transcription reaction containing 200 mM TrisHCl (pH 7.9), 30 mM MgCl_2_, 10 mM spermidine, 50 mM NaCl, 7.5 mM each of ATP, CTP, and GTP, 7 mM UTP, 0.5 mM Cy5-UTP (Cytiva), 2 μg of linearized template, 40 units RNasin (Promega), and 400 units of T7 RNA polymerase (Thermo-Fisher Scientific) (27, 31). The transcription reaction occurred at 37 °C for 4 h while mixing. The reaction products were run on a denaturing 7 M urea, 6% polyacrylamide gel at 20 W. The transcripts were visualized on the gel with shortwave UV light and excised from the gel. The excised product was eluted from the gel in 300 mM sodium acetate, 0.01% SDS, and 5 mM EDTA (pH 8) (31, 32). Ethanol precipitation was performed to concentrate the ACT1Δ6 pre-mRNA, and purity was assessed *via* absorbance values measured by a Nanodrop 2000C spectrophotometer and gel electrophoresis.

Splicing reactions were carried out in 60 mM potassium phosphate (pH 7), 2.5 mM MgCl_2_, 3% PEG (MW = 8000 amu), 1.5 nM ACT1Δ6 pre-mRNA, 0.4 units RNasin, and 4 μl of yeast splicing extract. The reactions either contained 10 mM ATP to allow splicing to proceed or 0.8% glucose as a negative control condition. Splicing extracts used in heat-shocked reactions were incubated at 38 °C for 18 min and then chilled on ice for 5 min. All splicing extracts were incubated at room temperature for 15 min prior to being added into splicing reactions. The splicing reactions proceeded at room temperature for 30 min. Stop buffer containing 1% SDS, 50 mM Tris (pH 8), 50 mM EDTA, and 2.5 mg/ml proteinase K was added at the end of the splicing reaction, and the solution was heated to 55 °C for 10 min for splicing inactivation (7, 27). Gel loading buffer containing 95% formamide, 0.025% bromophenol blue, and 5 mM EDTA (pH 8) was added to reaction mixtures, and then the samples were run on a 7 M urea, 6% denaturing polyacrylamide gel at 20 W. Spliced products were visualized by excitation at 650 nm on Typhoon FLA 9500 imager at 1000 PVT with 25 μm resolution (7). All *in vitro* splicing assays were conducted in triplicate with biological replicates.

### Dib1 protein purification

Rosetta (Novagen) *E. coli* cells were transformed with pET15b-*dib1* plasmids. The cells were heat-shocked at 42 °C to promote uptake of the plasmid. A single colony was inoculated overnight in LB with ampicillin (Amp, 100 μg/ml) and chloramphenicol (CAM, 25 μg/ml) and grown overnight at 37 °C and 250 RPM. Overnight cultures were inoculated and grown to an OD_600_ of 0.7. IPTG (1 mM) was added, and cells were incubated at 18 °C and 250 RPM for 16 to 18 h. Cell cultures were centrifuged at 5000 RPM for 10 min to pellet cells. The supernatant was removed, and the cells were resuspended in 10 mM imidazole buffer, 300 mM NaCl, 50 mM KPO_4_ (pH 7.6). The cell solution was sonicated with a QSonica Q700 sonicator at amplitude 60, process time 2 min, and pulse-ON/OFF times at 30 s with a microtip to lyse cells, and the lysate was centrifuged at 18,000 RPM for 45 min. The pellet was discarded, and the lysate was centrifuged again at 18,000 RPM for 30 min. The supernatant flowed through a Ni-NTA column (Fisher Scientific), which was equilibrated with 10 mM imidazole, 300 mM NaCl, 50 mM KPO_4_ (pH 7.6). The column was washed with increasing concentrations of imidazole (pH 7.6) on a gradient from 10 to 500 mM imidazole. Dib1 eluted at 250 mM imidazole. Fractions containing the protein eluent were then dialyzed in 50 mM Tris (pH 8.0), 100 mM NaCl, 1 mM DTT, and 5% glycerol. To remove the N-terminal His-tag, proteins were digested with thrombin (Sigma-Aldrich). Proteins were then dialyzed in a 20 mM Tris (pH 8.0), 10 mM NaCl, 1 mM DTT, and 5% glycerol buffer. The protein sample was flowed through Q-Sepharose resin (Cytiva) equilibrated with 20 mM Tris (pH 8.0) and 10 mM NaCl. The column was washed with increasing concentrations of NaCl at pH 8 on a gradient from 10 mM to 200 mM NaCl solutions. Fractions were visualized by SDS-PAGE gel, and fractions containing high concentrations of pure Dib1 protein were dialyzed in storage buffer containing 10 mM potassium phosphate (pH 7.6), 100 mM NaCl, 1 mM DTT, and 5% glycerol. Proteins were flash frozen in liquid nitrogen and stored at −80 °C.

### Time-of-flight mass spectrometry

Protein molecular masses were determined using an Agilent 6546 LC/Q-TOF-MS system. Purified protein samples were dialyzed against 10 mM phosphate buffer (pH 7.6) with 100 mM NaCl. After dialysis, protein concentration was measured on a Nanodrop 2000C by absorbance at 280 nm, and all samples were diluted to 50 μM. The Agilent PLRP-S column was used for chromatographic separation of the Dib1 samples. The column was equilibrated for 5 min with 90% acetonitrile/0.1% formic acid in water. Chromatographic separation of each protein sample was successfully achieved by two successive gradient elutions from 0.1% formic acid to 0.1% formic acid/90% acetonitrile, which was followed by methanol washes to remove residual protein on the column. The oven was heated to 70 °C with dual ESI 220 V positive mode ion source. Data were analyzed using Agilent BioConfirm through integration and extraction of the elution peak, followed by deconvolution of each mass-to-charge ratio to obtain a single molecular weight.

### Circular dichroism denaturing assays

Recombinant Dib1 proteins were dialyzed in 10 mM phosphate buffer pH 8.0 with 100 mM NaCl to remove glycerol from the storage buffer. CD samples were prepared at a final protein concentration of 9 μM Dib1 and incubated with increasing concentrations of guanidinium hydrochloride (GdnHCl). Dib1 protein samples reacted with the denaturant for 1 hour on ice and were then measured on a JASCO J-815 CD spectrometer. Full spectra were taken from 195 nm to 240 nm at 25 °C with a data pitch of 1 nm, 50 nm/min speed, 2 s response, 10 nm bandwidth with six accumulations in continuous scanning mode. At the wavelength where the 0 M GdnHCl sample exhibited the largest magnitude of ellipticity (220 nm), the ellipticity was plotted against the [GdnHCl] to produce a denaturation curve. Graphpad Prism 10 was used to analyze the curve by fitting a nonlinear regression with four parameters. The experimental denaturation data of recombinant Dib1 follows the Hill equation (30):

$${y=y}_{0}+\frac{ax^{b}}{c^{b}+x^{b}}$$

where y is the measured ellipticity, $y_{0}$ is the baseline value of the lowest [GdnHCl], a is the difference between y_max_ and $y_{0}$, x is [GdnHCl], b is the Hill coefficient, and c is the effective [GdnHCl] at which 50% of recombinant Dib1 has denatured. The fraction of denatured recombinant Dib1 at any [GdnHCl] can be calculated according to the following (30):

$$f_{D}=\frac{y-y_{0}}{y_{\max}-y_{0}}$$

The equilibrium constant of denaturation for any [GdnHCl] can be calculated from the fraction of protein denaturation according to the following (33):

$$K_{D}=\frac{f_{D}}{1-f_{D}}$$

The free energy of unfolding for each recombinant protein at any [GdnHCl] can be calculated through (33):

$$\Delta G=-RTln\left(K_{D}\right)$$

All CD trials were completed a minimum of three times per protein mutant.

## Data availability

All data are contained in the manuscript and supplemental information.

## Supporting information

This article contains supporting information.

## Conflict of interest

The authors declare that they have no conflicts of interest with the contents of this article.
