# Supplementary material for: The evolutionarily conserved C-terminal tail of the Dib1 protein is required for spliceosome efficacy and Dib1 protein stability
Source: J Biol Chem. 2026 Aug 5;302(9):113395. doi: 10.1016/j.jbc.2026.113395 (PMC13560316; doi:10.1016/j.jbc.2026.113395)
Supplement: Supplementary file 1 [file mmc1.pdf]

Supplemental Information

Supplemental Figure 1. Sequence alignment of conserved Dib1 amino acid sequences across six species, including *S. cerevisiae* and *H. sapiens.* The amino acid sequences were aligned and visualized using the Jalview Coffee T tool.

Supplemental Figure 2. Deconvoluted mass spectra of all purified protein samples used in these studies. The Dib1 protein variant and theoretical molecular weight are included in the top left corner of each mass spectra. Mass spectra were obtained using the Agilent 6546 LC/Q-TOF-MS system, and primary data was deconvoluted using BioConfirm software.

Supplemental Figure 3. Extrapolation plots for the $\Delta G_{unfolding}$ at 0 M denaturant for Dib1 full length, Dib1Δ3, Dib1Δ6, and Dib1Δ9 proteins. Individual trials are shown as unique lines with varying darkness. The individual points on the plots are plotted using the equations in the materials and methods, and a line of best fit was plotted for the extrapolation. The slopes of these extrapolation plots are displayed in Supplementary Table 1. The R^2 values of all lines are between 0.9994 and 0.9986.

Supplemental Figure 4. Extrapolation plots for the $\Delta G_{unfolding}$ at 0 M denaturant for Dib1Δ12, Dib1Δ13, Dib1Δ14, and Dib1Δ15 proteins. Individual trials are shown as unique lines with varying darkness. The individual points on the plots are plotted using the equations in the materials and methods, and a line of best fit was plotted for the extrapolation. The slopes of these extrapolation plots are displayed in Supplementary Table 1. The R^2 values of all lines are between 0.9994 and 0.9986.

Supplemental Figure 5. Extrapolation plots for the $\Delta G_{unfolding}$ at 0 M denaturant for Dib1Δ16, Dib1Δ14-G129A, and Dib1Δ15-K128A proteins. Individual trials are shown as unique lines with varying darkness. The individual points on the plots are plotted using the equations in the materials and methods, and a line of best fit was plotted for the extrapolation. The slopes of these extrapolation plots are displayed in Supplementary Table 1. The R^2 values of all lines are between 0.9994 and 0.9986.

Supplementary Figure 1.

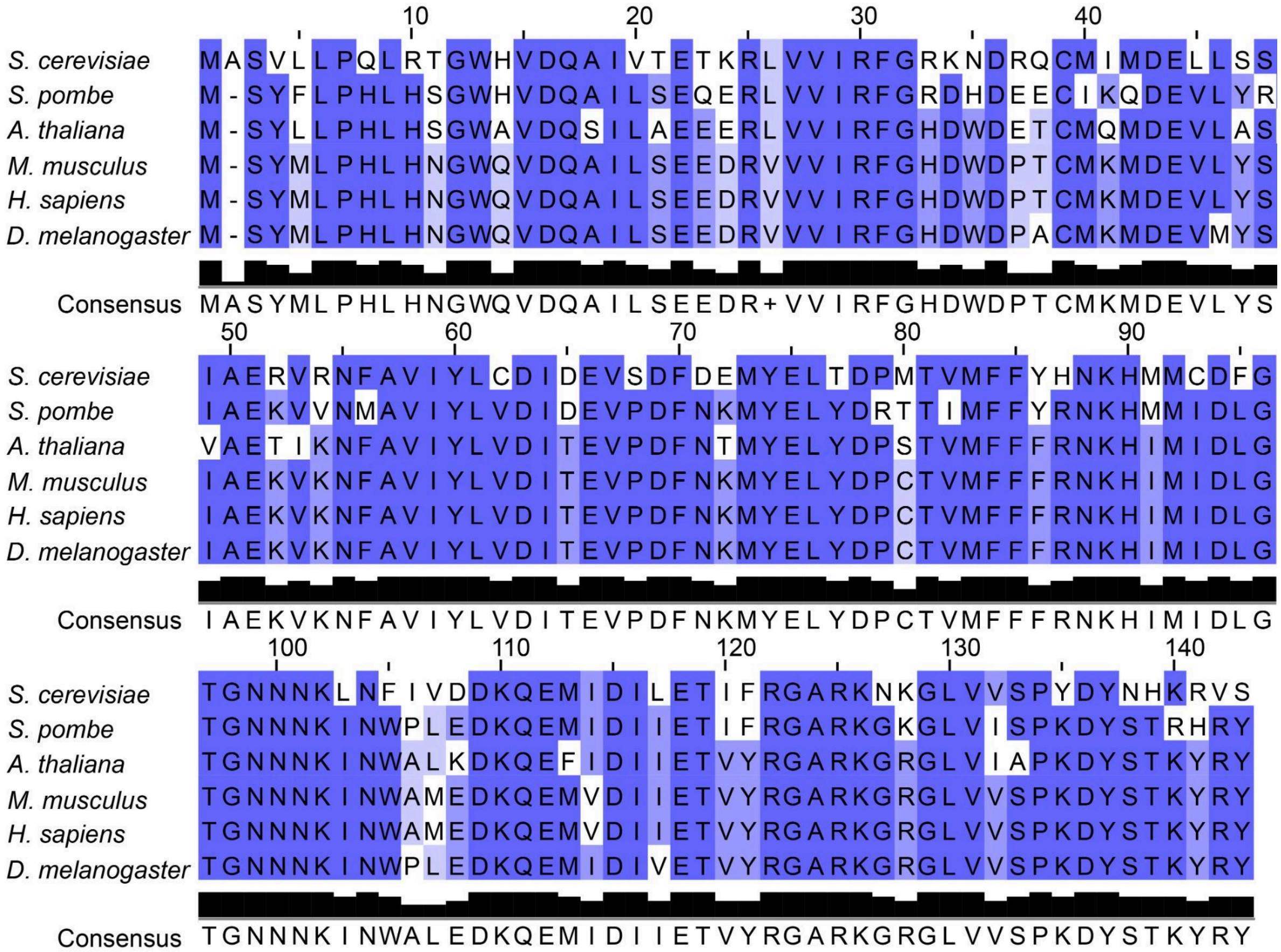

Supplementary Figure 2.

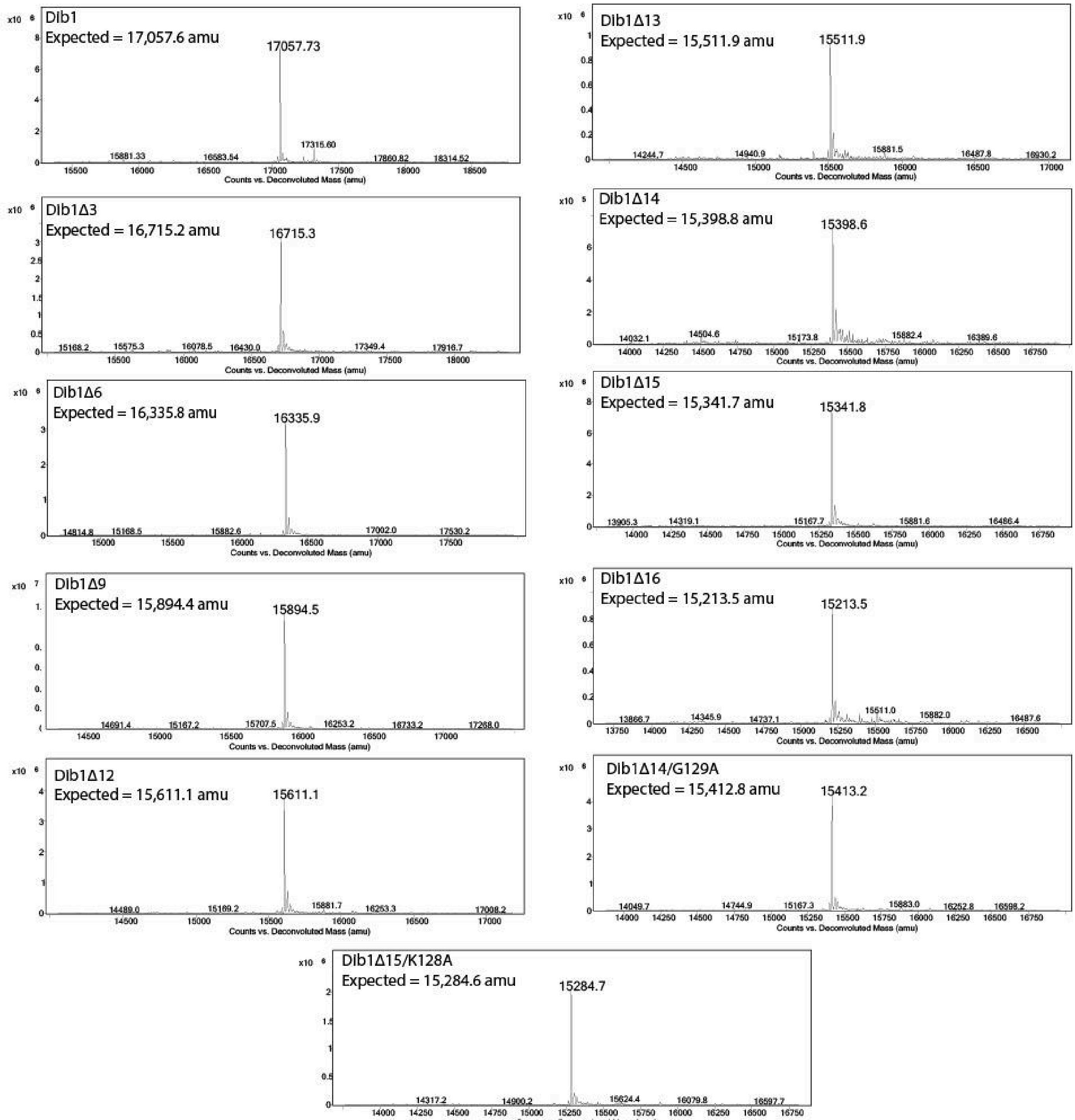

Supplementary Figure 3.

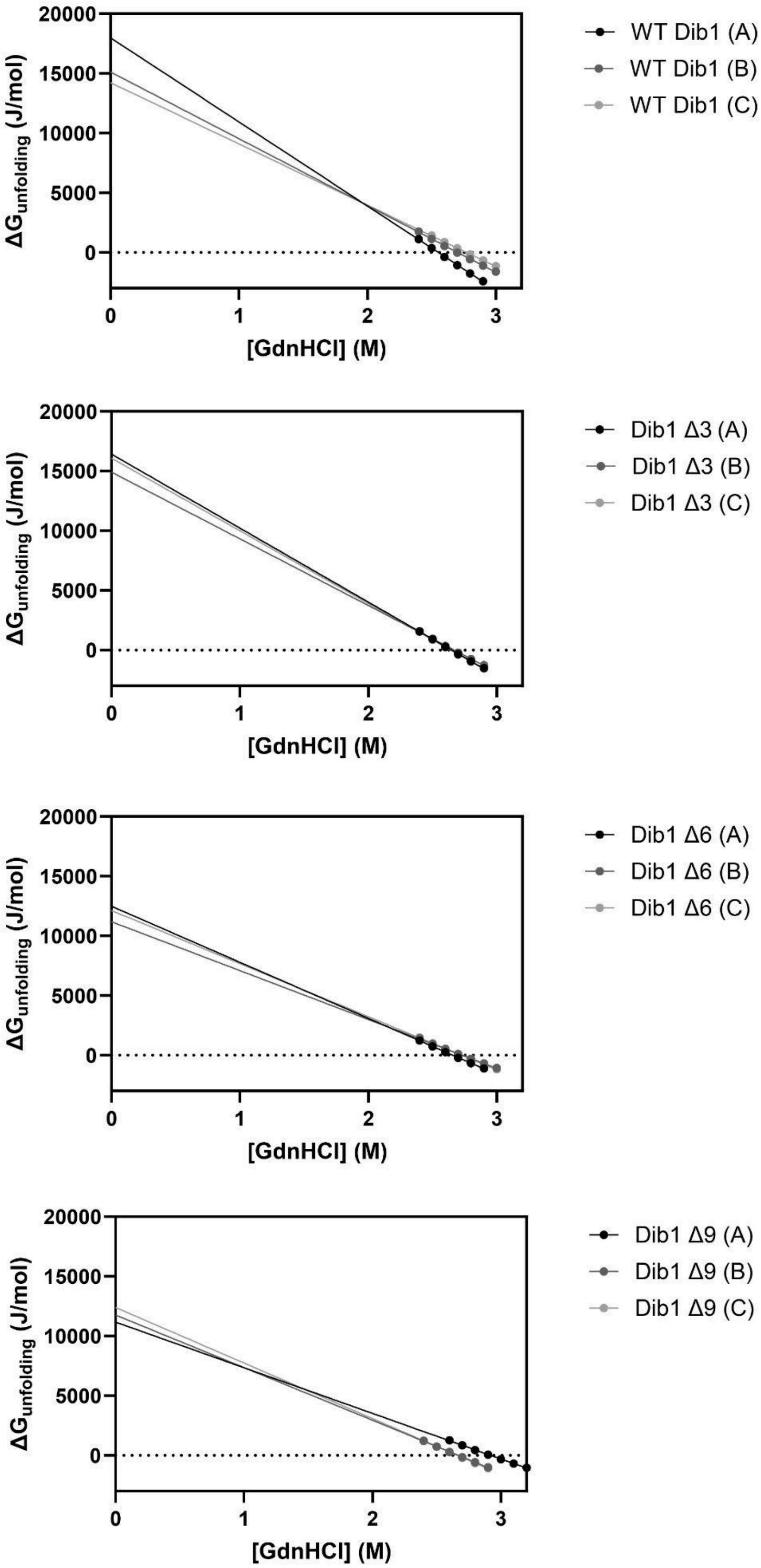

Supplementary Figure 4.

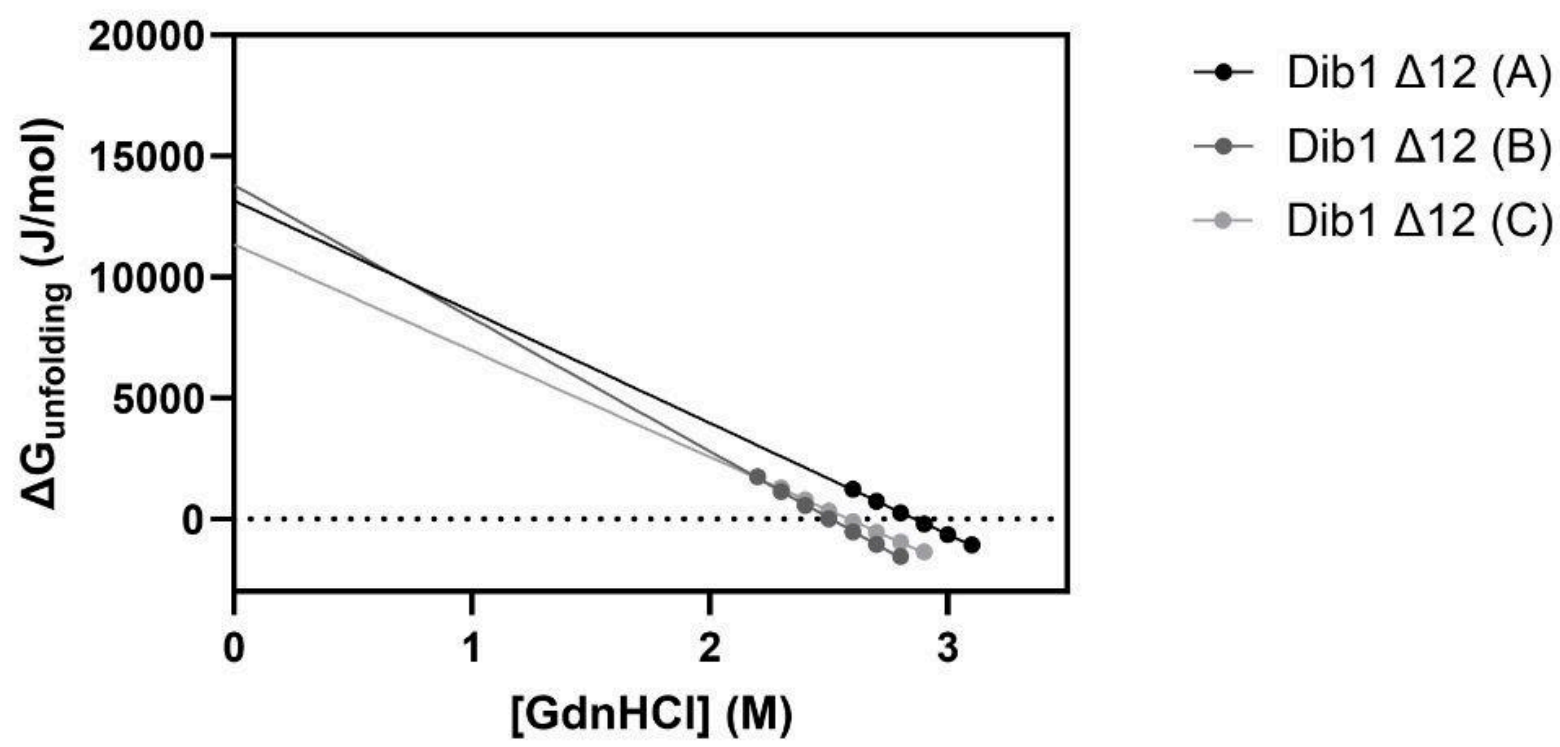

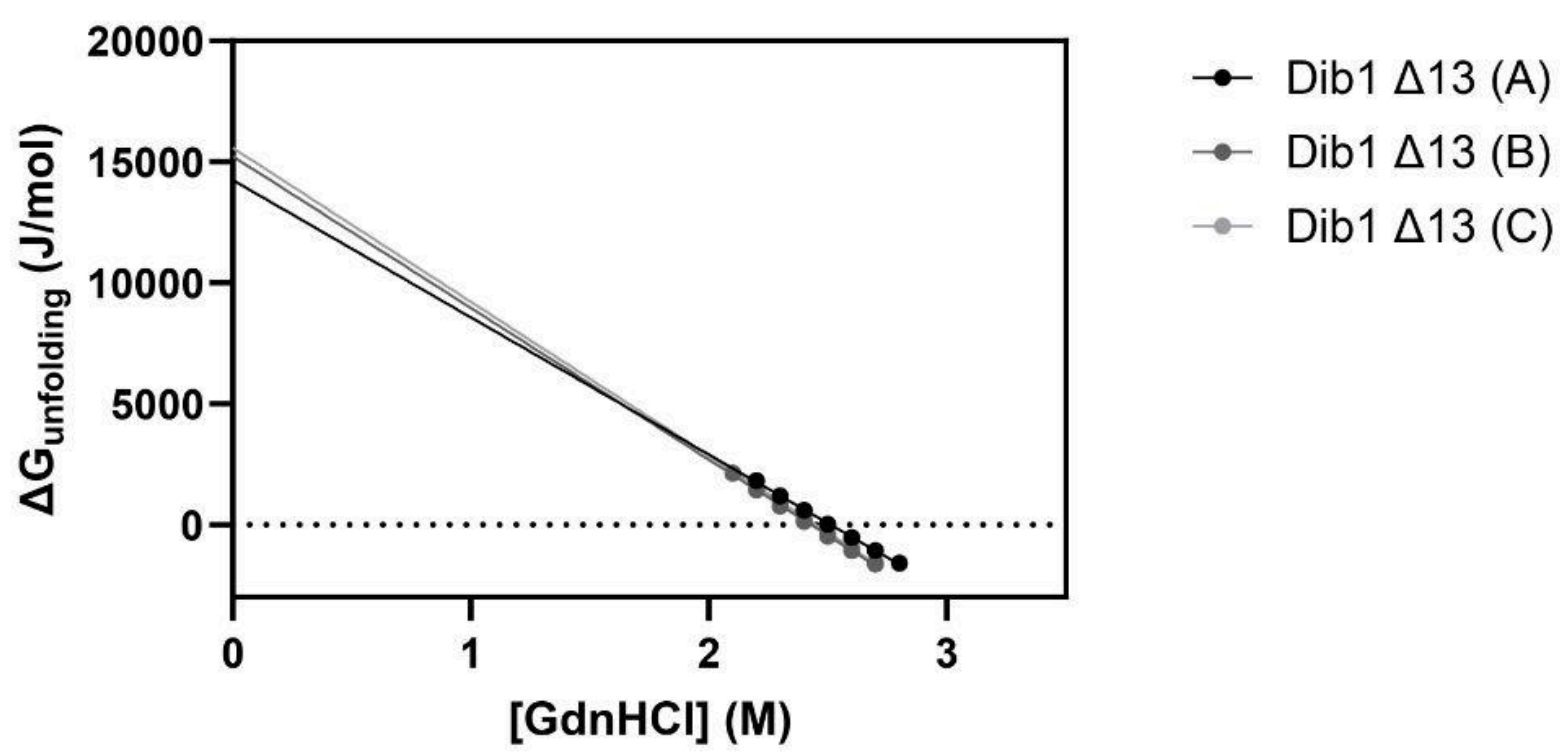

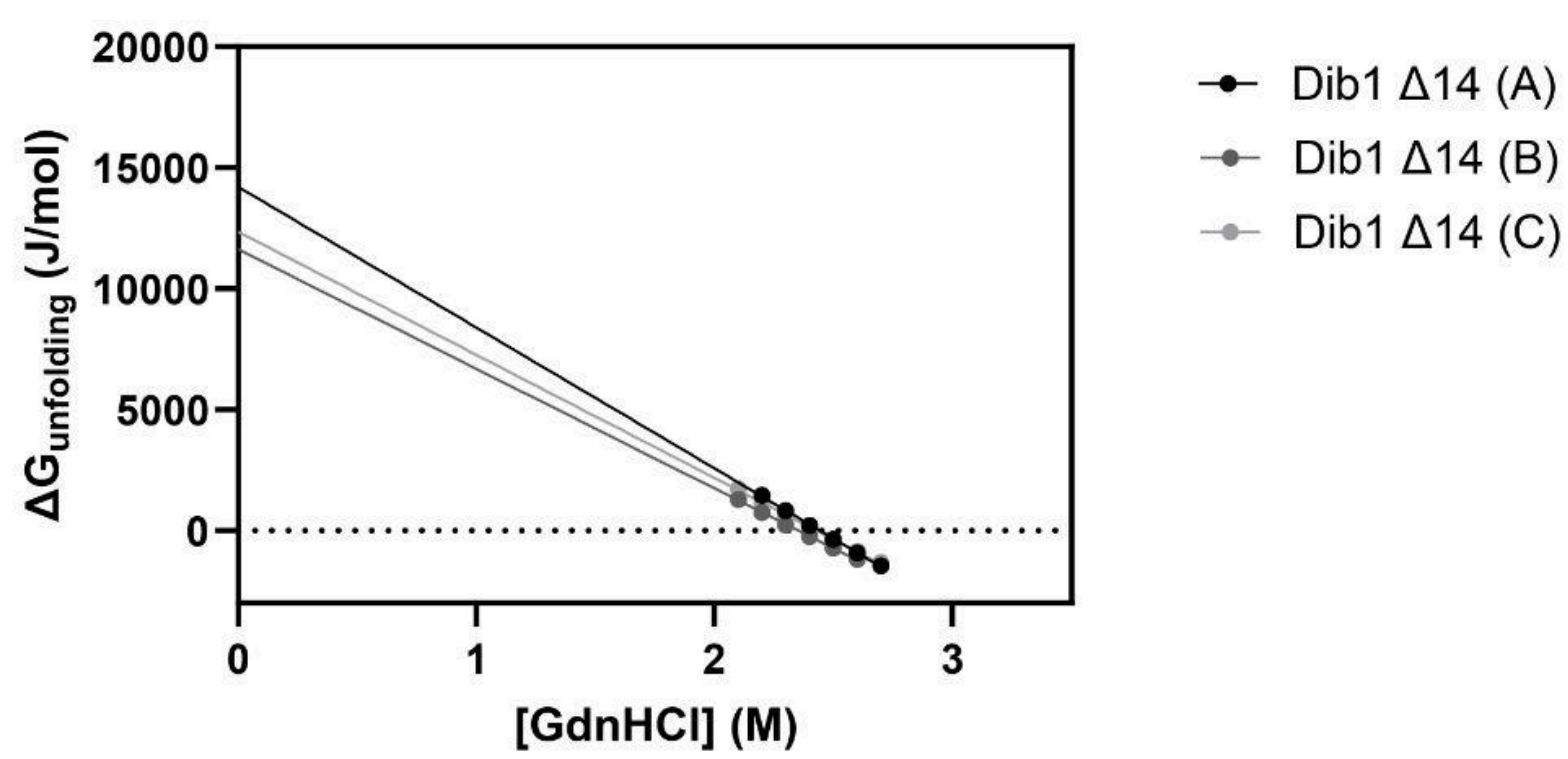

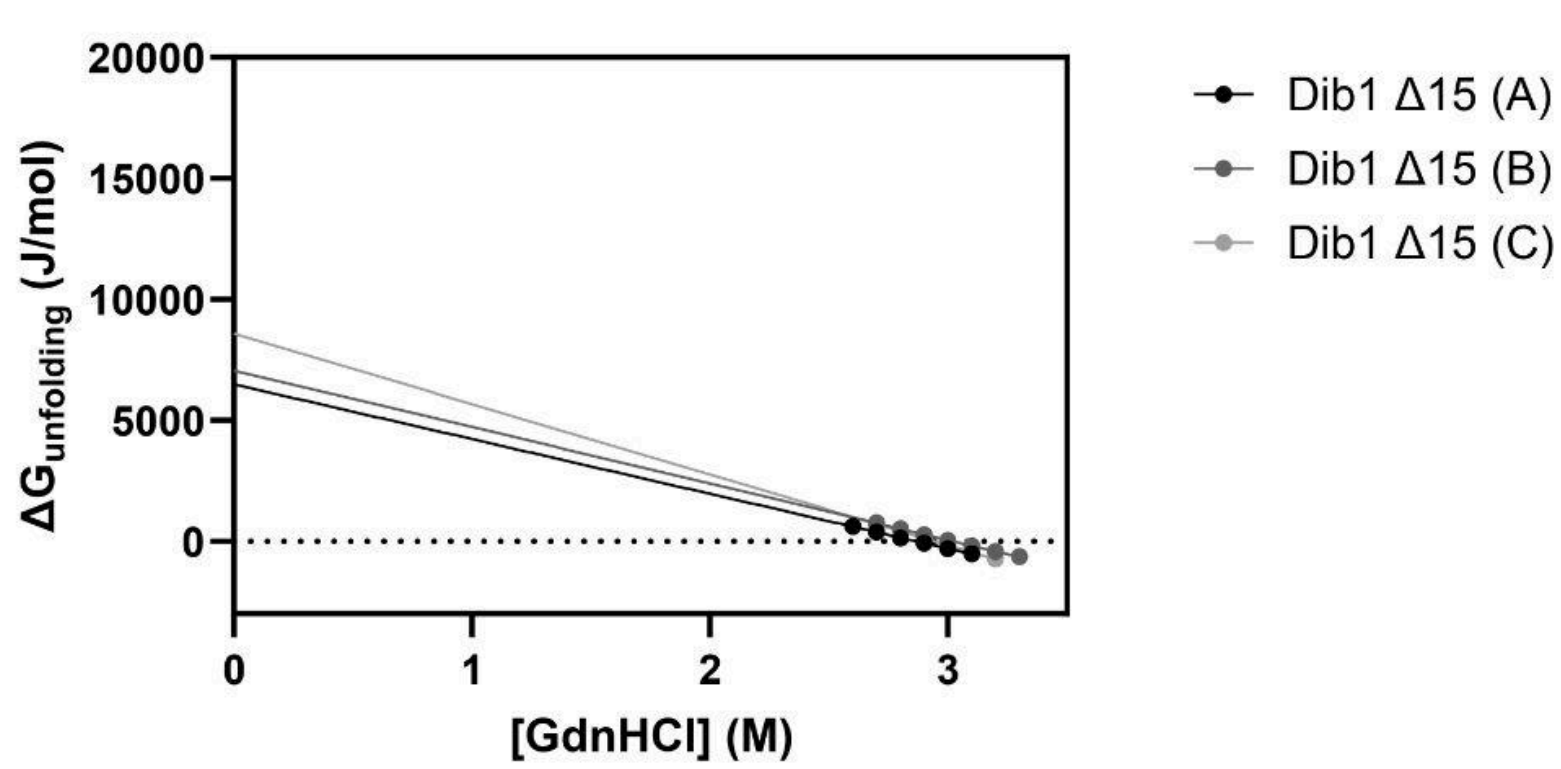

Supplementary Figure 5.

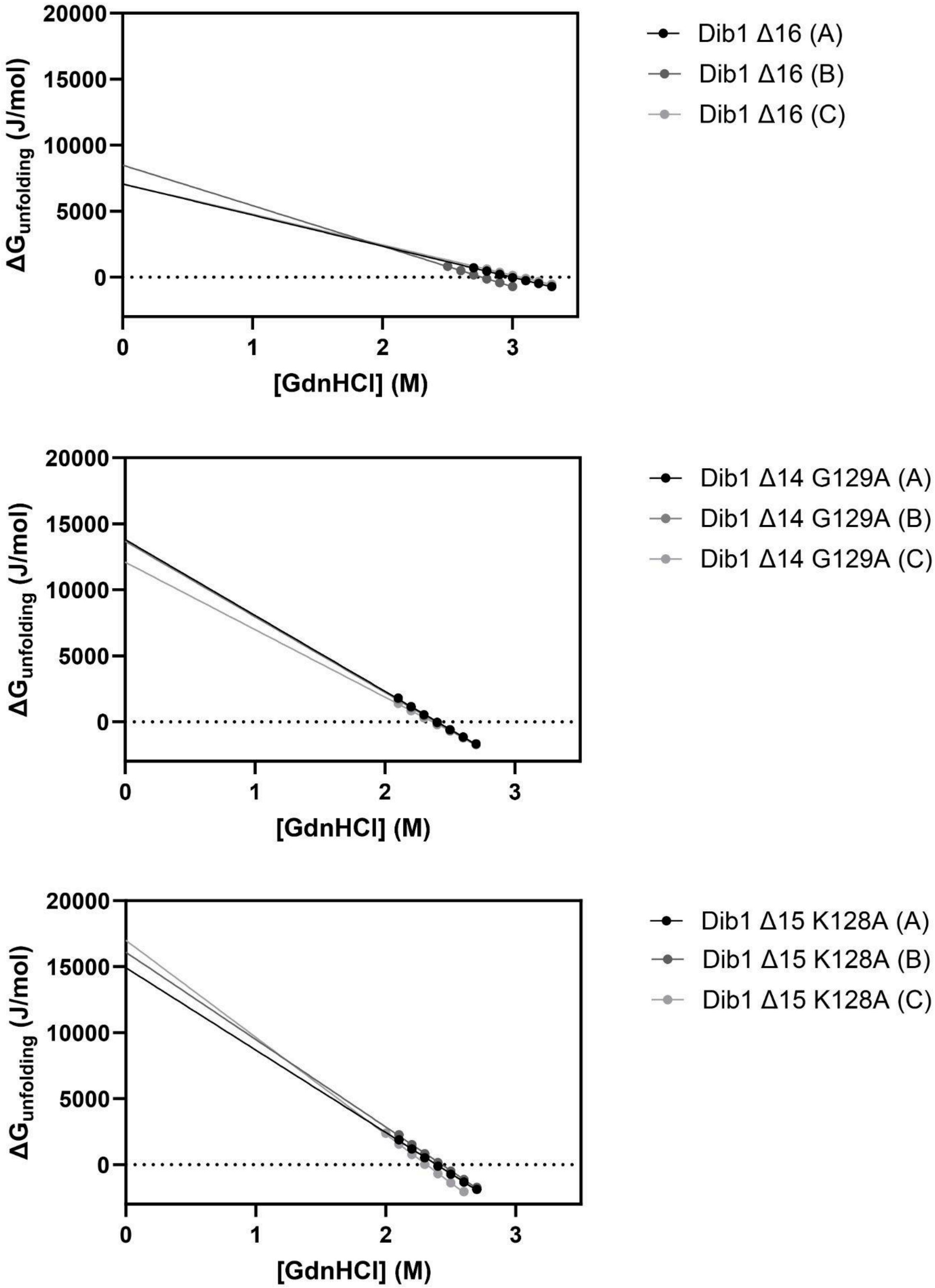

Table S1. The average trendline slope of the delta G extrapolation plots in Supplementary Figure 3. All values are based on three trials.

Protein	Slope (m-value) ($\frac{kJ/mol}{M\ (GdnHCl)}$)	Molecular Weight (amu)
Dib1	-5.93 ± 1.00	17,057
Dib1Δ3	-5.96 ± 0.33	16,715
Dib1Δ6	-4.41 ± 0.30	16,335
Dib1Δ9	-4.29 ± 0.43	15,894
Dib1Δ12	-4.84 ± 0.59	15,610
Dib1Δ13	-6.10 ± 0.37	15,511
Dib1Δ14	-5.28 ± 0.47	15,398
Dib1Δ15	-2.51 ± 0.38	15,341
Dib1Δ16	-2.58 ± 0.43	15,213
Dib1Δ14 G129A	-5.53 ± 0.36	15, 413
Dib1Δ15 K128A	-6.74 ± 0.56	15,284

Table S2. Oligonucleotides used for yeast expression in this study.

Name	Sequence (5'–3')	Description
**oCM310**	GTGGTCTCTCCATACGATTATAATCATAAGTAATTCATAATAACTATGTAGGCTAGGCGC	Forward DIB1Δ3 C-terminus truncation for yeast expression (ΔRVS) ‡
**oCM311**	GCGCCTAGCCTACATAGTTATTATGAATTACTTATGATTATAATCGTATGGAGAGACCAC	Reverse DIB1Δ3 C-terminus truncation for yeast expression (ΔRVS)
**oCM312**	GGGCTGGTGGTCTCTCCATACGATTATTAATTCATAATAACTATGTAGGCT	Forward DIB1Δ6 C-terminus truncation for yeast (ΔNHKRVS)
**oCM313**	AGCCTACATAGTTATTATGAATTAATAATCGTATGGAGAGACCACCAGCCC	Reverse DIB1Δ6 C-terminus truncation for yeast (ΔNHKRVS)

**oCM314**	AAAGGGCTGGTGGTCTCTCCATAATTCATAATAACTATGTAGGCT	Forward DIB1Δ9 C-terminus truncation for yeast (ΔYDYNHKRVS)
**oCM315**	GGGCTGGTGGTCTCTCCATACGATTATTAATTCATAATAACTATGTAGGCT	Reverse DIB1Δ9 C-terminus truncation for yeast (ΔYDYNHKRVS)
**oCM376**	CCAGGAAGAACAAAGGGCTGGTGTAATTCATAATAACTATGTAGGCT	Forward DIB1Δ12 C-terminus truncation for yeast (ΔVSPYDYNHKRVS)
**oCM377**	AGCCTACATAGTTATTATGAATTACACCAGCCCTTTGTTCTTCCTGG	Reverse DIB1Δ12 C-terminus truncation for yeast (ΔVSPYDYNHKRVS)
**oCM594**	GCCAGGAAGAACAAAGGGCTGTAATTCATAATAACTATGTAGG	Forward DIB1Δ13 C-terminus truncation for yeast (ΔVVSPYDYNHKRVS)
**oCM595**	CCTACATAGTTATTATGAATTACAGCCCTTTGTTCTTCCTGGC	Reverse DIB1Δ13 C-terminus truncation for yeast (ΔVVSPYDYNHKRVS)
**oCM390**	AGAGGTGCCAGGAAGAACAAAGGGTAATTCATAATAACTATGTAGGCT	Forward DIB1Δ14 C-terminus truncation for yeast (ΔLVVSPYDYNHKRVS)
**oCM391**	AGCCTACATAGTTATTATGAATTACCCTTTGTTCTTCCTGGCACCTCT	Reverse DIB1Δ14 C-terminus truncation for yeast (ΔLVVSPYDYNHKRVS)
**oCM378**	GACATATTAGAAACTATATTCAGAGGTGCCAGGAAGAACAAATAATTCATAATAACTATGTAGGCT	Forward DIB1Δ15 C-terminus truncation for yeast (ΔGLVVSPYDYNHKRVS)
**oCM379**	AGCCTACATAGTTATTATGAATTATTTGTTCTTCCTGGCACCTCTGAATATAGTTTCTAATATGTC	Reverse DIB1Δ15 C-terminus truncation for yeast (ΔGLVVSPYDYNHKRVS)
**oCM388**	GGTGCCAGGAAGAACTAATTCATAATAACTATGTAGGCTAGGCGC	Forward DIB1Δ16 C-terminus truncation for yeast (ΔKGLVVSPYDYNHKRVS)
**oCM389**	GCGCCTAGCCTACATAGTTATTATGAATTAGTTCTTCCTGGCACC	Reverse DIB1Δ16 C-terminus truncation for yeast (ΔKGLVVSPYDYNHKRVS)
**oCM698**	TAATCGTATGGAGAGACCGCCGCCCCTTTGTTCTTCCTGGC	Forward DIB1 L130A/V131A for yeast
**oCM699**	GCCAGGAAGAACAAAGGGGCGGCGGTCTCTCCATACGATTA	Reverse DIB1 L130A/V131A for yeast
**oCM700**	CATAGTTATTATGAATTACACCGCCCCTTTGTTCTTCCTGGCACC	Forward DIB1Δ12 C-terminus truncation and L130A for yeast (**L**VΔVSPYDYNHKRVS)*

**oCM701**	GGTGCCAGGAAGAACAAAGGGGCG GTGTAATTCATAATAACTATG	Reverse DIB1Δ12 C-terminus truncation and L130A for yeast (**L**VΔVSPYDYNHKRVS)
**oCM702**	CTACATAGTTATTATGAATTACGCCA GCCCTTTGTTCTTCCTGG	Forward DIB1Δ12 C-terminus truncation and V131A for yeast (L**V**ΔVSPYDYNHKRVS)
**oCM703**	CCAGGAAGAACAAAGGGCTGGCGT AATTCATAATAACTATGTAG	Reverse DIB1Δ12 C-terminus truncation and V131A for yeast (L**V**ΔVSPYDYNHKRVS)
**oCM704**	GGTGCCAGGAAGAACAAAGGGGCG GCGTAATTCATAATAACTATGTAG	Forward DIB1Δ12 C-terminus truncation and L130A/V131A for yeast (**LV**ΔVSPYDYNHKRVS)
**oCM705**	CTACATAGTTATTATGAATTACGCCG CCCCTTTGTTCTTCCTGGCACC	Reverse DIB1Δ12 C-terminus truncation and L130A/V131A for yeast (**LV**ΔVSPYDYNHKRVS)
**oCM668**	CAGAGGTGCCAGGAAGAACAAAGC TTAATTCATAATAACTATGTAGGC	Forward DIB1Δ14 C-terminus truncation and G129A for yeast (**G**ΔLVVSPYDYNHKRVS)
**oCM669**	GCCTACATAGTTATTATGAATTAAGC TTTGTTCTTCCTGGCACCTCTG	Reverse DIB1Δ14 C-terminus truncation and G129A for yeast (**G**ΔLVVSPYDYNHKRVS)
**oCM674**	ACTATATTCAGAGGTGCCAGGAAGA ACGCGTAATTCATAATAACTATGTAG GCTAGG	Forward DIB1Δ15 C-terminus truncation and K128A for yeast (**K**ΔGLVVSPYDYNHKRVS)
**oCM675**	CCTAGCCTACATAGTTATTATGAATTA CGCGTTCTTCCTGGCACCTCTGAATA TAGT	Reverse DIB1Δ15 C-terminus truncation and K128A for yeast (**K**ΔGLVVSPYDYNHKRVS)

‡ Δ represents the number and identity of residues truncated from the C-terminus of Dib1.
*bolded residues represent the desired alanine substitution.

Table S3. Oligonucleotides used for recombinant protein expression in this study.

**Name**	**Sequence (5'–3')**	**Description**
**oCM304**	ATACGATTATAATCATAAGTAAAG ATCCGGCTGCTAACAAAG	Forward primer for Dib1Δ3 C-terminus truncation for protein expression (ΔRVS‡)
**oCM305**	TATGCTAATATTAGTATTCATTTCT AGGCCGACGATTGTTTC	Reverse primer for Dib1Δ3 C-terminus truncation for protein expression (ΔRVS)
**oCM306**	GTGGTCTCTCCATACGATTATTAA AGATCCGGCTGCTAACAA	Forward primer for Dib1Δ6 C-terminus truncation for protein expression (ΔNHKRVS)

**oCM307**	CACCAGAGAGGTATGCTAATAATTTCTAGGCCGACGATTGTT	Reverse primer for Dib1Δ6 C-terminus truncation for protein expression (ΔNHKRVS)
**oCM308**	CTGGTGGTCTCTCCATAAAGATCCGGCTGCTAA	Forward primer for Dib1Δ9 C-terminus truncation for protein expression (ΔYDYNHKRVS)
**oCM309**	GACCACCAGAGAGGTATTTCTAGGCCGACGATT	Reverse primer for Dib1Δ9 C-terminus truncation for protein expression (ΔYDYNHKRVS)
**oCM380**	AAAGGGCTGGTGTAAAGATCCGGCTGCTAA	Forward primer for Dib1Δ12 C-terminus truncation for protein expression (ΔVSPYDYNHKRVS)
**oCM381**	TTTCCCGACCACATTTCTAGGCCGACGATT	Reverse primer for Dib1Δ12 C-terminus truncation for protein expression (ΔVSPYDYNHKRVS)
**oCM596**	GGAAGAACAAAGGGCTGTAAAGATCCGGCTGCTA	Forward primer for Dib1Δ13 C-terminus truncation for protein expression (ΔVVSPYDYNHKRVS)
**oCM597**	AGGAAGAACAAAGGGTAAAGATCCGGCTGCTAACAA	Reverse primer for Dib1Δ13 C-terminus truncation for protein expression (ΔVVSPYDYNHKRVS)
**oCM382**	AGGAAGAACAAAGGGTAAAGATCCGGCTGCTAACAA	Forward primer for Dib1Δ14 C-terminus truncation for protein expression (ΔLVVSPYDYNHKRVS)
**oCM383**	TCCTTCTTGTTTCCCATTTCTAGGCCGACGATTGTT	Reverse primer for Dib1Δ14 C-terminus truncation for protein expression (ΔLVVSPYDYNHKRVS)
**oCM384**	GGTGCCAGGAAGAACAAATAAAGATCCGGCTGCTAA	Forward primer for Dib1Δ15 C-terminus truncation for protein expression (ΔGLVVSPYDYNHKRVS)
**oCM385**	CCACGGTCCTTCTTGTTTATTTCTAGGCCGACGATT	Reverse primer for Dib1Δ15 C-terminus truncation for protein expression (ΔGLVVSPYDYNHKRVS)
**oCM386**	GGTGCCAGGAAGAACTAAAGATCCGGCTGCTAA	Forward primer for Dib1Δ16 C-terminus truncation for protein expression (ΔKGLVVSPYDYNHKRVS)

**oCM387**	CCACGGTCCTTCTTGATTTCTAGG CCGACGATT	Reverse primer for Dib1Δ16 C-terminus truncation for protein expression (ΔKGLVVSPYDYNHKRVS)
**oCM698**	TAATCGTATGGAGAGACCGCCGCC CCTTTGTTCTTCCTGGC	Forward Dib1 L130A/V131A for protein expression
**oCM699**	GCCAGGAAGAACAAAGGGGCGG CGGTCTCTCCATACGATTA	Reverse Dib1 L130A/V131A for protein expression
**oCM706**	CCAGGAAGAACAAAGGGGCGGT GTAAAGATCCGGCT	Forward Dib1Δ12 C-terminus truncation and L130A for protein expression (**L**VΔVSPYDYNHKRVS)*
**oCM707**	AGCCGGATCTTTACACCGCCCCTT TGTTCTTCCTGG	Reverse Dib1Δ12 C-terminus truncation and L130A for protein expression (**L**VΔVSPYDYNHKRVS)
**oCM708**	AAGAACAAAGGGCTGGCGTAAA GATCCGGCTGC	Forward Dib1Δ12 C-terminus truncation and V131A for protein expression (L**V**ΔVSPYDYNHKRVS)
**oCM709**	GCAGCCGGATCTTTACGCCAGCC CTTTGTTCTT	Reverse Dib1Δ12 C-terminus truncation and V131A for protein expression (L**V**ΔVSPYDYNHKRVS)
**oCM710**	CCAGGAAGAACAAAGGGGCGGC GTAAAGATCCGGCTGCT	Forward Dib1Δ12 C-terminus truncation and L130A/V131A for protein expression (**LV**ΔVSPYDYNHKRVS)
**oCM711**	AGCAGCCGGATCTTTACGCCGCC CCTTTGTTCTTCCTGG	Reverse Dib1Δ12 C-terminus truncation and L130A/V131A for protein expression (**LV**ΔVSPYDYNHKRVS)
**oCM670**	GCCAGGAAGAACAAAGCGTAAA GATCCGGCTGC	Forward Dib1Δ14 C-terminus truncation and G129A for protein expression (**G**ΔLVVSPYDYNHKRVS)
**oCM671**	GCAGCCGGATCTTTACGCTTTGTT CTTCCTGGC	Reverse Dib1Δ14 C-terminus truncation and G129A for protein expression (**G**ΔLVVSPYDYNHKRVS)
**oCM672**	TCAGAGGTGCCAGGAAGAACGCG TAAAGATCCGGCTGCCAAC	Forward Dib1Δ15 C-terminus truncation and K128A for protein expression (**K**ΔGLVVSPYDYNHKRVS)

**oCM673**	GTTGGCAGCCGGATCTTTACGCGT TCTTCCTGGCACCTCTGA	Reverse Dib1Δ15 C-terminus truncation and K128A for protein expression (**K**ΔGLVVSPYDYNHKRVS)

‡ Δ represents the number and identity of residues truncated from the C-terminus of Dib1.
*bolded residues represent the desired alanine substitution.
